# SNP discovery and genetic mapping using genotyping by sequencing of whole genome genomic DNA from a pea RIL population

**DOI:** 10.1186/s12864-016-2447-2

**Published:** 2016-02-18

**Authors:** Gilles Boutet, Susete Alves Carvalho, Matthieu Falque, Pierre Peterlongo, Emeline Lhuillier, Olivier Bouchez, Clément Lavaud, Marie-Laure Pilet-Nayel, Nathalie Rivière, Alain Baranger

**Affiliations:** INRA, UMR 1349 IGEPP, BP35327, Le Rheu Cedex, 35653 France; INRA, UMR Génétique Quantitative et Evolution – Le Moulon, INRA - Univ Paris-Sud - CNRS - AgroParisTech, Ferme du Moulon, 91190 Gif-sur-Yvette, France; INRIA Rennes - Bretagne Atlantique/IRISA, EPI GenScale, Rennes, 35042 France; GeT-PlaGe, Genotoul, INRA Auzeville F31326, Castanet-tolosan, France; Biogemma, route d’Ennezat, CS90126, Chappes, 63720 France; INRA, UMR1388 INRA/ENVT/ENSAT GenPhySE, INRA Auzeville F31326, Castanet-tolosan, France; PISOM, UMT INRA/CETIOM, BP35327, Le Rheu Cedex, 35653 France

**Keywords:** *Aphanomyces euteiches*, *Pisum sativum*, Next generation sequencing, Marker assisted selection, QTL

## Abstract

**Background:**

Progress in genetics and breeding in pea still suffers from the limited availability of molecular resources. SNP markers that can be identified through affordable sequencing processes, without the need for prior genome reduction or a reference genome to assemble sequencing data would allow the discovery and genetic mapping of thousands of molecular markers. Such an approach could significantly speed up genetic studies and marker assisted breeding for non-model species.

**Results:**

A total of 419,024 SNPs were discovered using HiSeq whole genome sequencing of four pea lines, followed by direct identification of SNP markers without assembly using the discoSnp tool. Subsequent filtering led to the identification of 131,850 highly designable SNPs, polymorphic between at least two of the four pea lines.

A subset of 64,754 SNPs was called and genotyped by short read sequencing on a subpopulation of 48 RILs from the cross ‘Baccara’ x ‘PI180693’. This data was used to construct a WGGBS-derived pea genetic map comprising 64,263 markers. This map is collinear with previous pea consensus maps and therefore with the *Medicago truncatula* genome. Sequencing of four additional pea lines showed that 33 % to 64 % of the mapped SNPs, depending on the pairs of lines considered, are polymorphic and can therefore be useful in other crosses.

The subsequent genotyping of a subset of 1000 SNPs, chosen for their mapping positions using a KASP™ assay, showed that almost all generated SNPs are highly designable and that most (95 %) deliver highly qualitative genotyping results. Using rather low sequencing coverages in SNP discovery and in SNP inferring did not hinder the identification of hundreds of thousands of high quality SNPs.

**Conclusions:**

The development and optimization of appropriate tools in SNP discovery and genetic mapping have allowed us to make available a massive new genomic resource in pea. It will be useful for both fine mapping within chosen QTL confidence intervals and marker assisted breeding for important traits in pea improvement.

**Electronic supplementary material:**

The online version of this article (doi:10.1186/s12864-016-2447-2) contains supplementary material, which is available to authorized users.

## Background

SNPs (Single Nucleotide Polymorphisms) are genetic markers of choice for both linkage and association mapping and for population structure and evolution analysis. They are virtually unlimited, evenly distributed along the genome, bi-allelic and co-dominant. Massive SNP discovery was first limited to the few species with an available reference genome. Recently, with the advances in cheaper next generation sequencing (NGS) technologies, various accessions within species even with complex genomes could be sequenced [1]. The challenge of sequencing large genomes with high levels of repeated sequences first led to the development of novel approaches for reducing genome complexity [2]. cDNA sequencing, which specifically addresses the expressed genic fraction, was largely developed and reviewed in Duarte et al. [3]. Restriction site Associated DNA (RAD) tags have been applied to a large range of organisms such as *Drosophila melanogaster* [4], fish and fungi [5]. In plants, RAD-Seq has been applied to a number of species for both large-scale SNP discovery and the mapping of SNP subsets in barley [6] and rye-grass [7]. In legume species, Deokar et al. [8] first reported the use of RAD-Seq in chickpea to discover 29,000 SNPs and subsequently map 604 recombination bins. Restriction enzyme digest to reduce genome complexity followed by direct Genotyping-by-Sequencing was reported for maize RILs and barley doubled haploid lines [9], where 2,382 markers were eventually mapped on the barley genetic map. In legume species, Sonah et al. [10] first used GBS in soybean to develop 10,120 high quality SNPs. Thus all these studies used genome reduction and various assembling tools.

Pea is the third production in the world among temperate grain legume crops after soybean and common bean and is a major source of protein for humans and livestock. Pea is particularly relevant in temperate cropping systems due to its capacity to fix nitrogen through symbiosis. Nevertheless, the species suffers from significant yield instability due to its high susceptibility to abiotic and biotic stresses, among which Aphanomyces root rot disease, due to the oomycete *Aphanomyces euteiches* Drechs. Resistance Quantitative Trait Loci (QTL) have been described, but the QTL confidence intervals are still large, especially due to the lack of markers and low resolution of existing genetic maps. It remains a challenge to reduce QTL confidence intervals, to discover underlying candidate genes and develop breeding programs using molecular markers strongly associated with phenotypes.

Although pea has actually entered the genomic era [11], it still suffers from limited genomic resources compared to other crops. The pea genome is 4.3 Gb, which is around 10 times larger than the genome of the model species *M.truncatula* [12]. This includes repeats mostly derived from transposon-based sequences [13]. Recent reports indicated that large new sequencing resources are under development [14] and that a consortium for pea genome sequencing is at work (http://www.coolseasonfoodlegume.org/pea_genome), however no full genome sequence is available yet. Large numbers of new molecular markers are still needed to saturate pea maps and significantly improve QTL mapping both for research and breeding objectives. Although transcriptome sequencing has recently been used in pea for SNP discovery [3, 15, 16] and mapping [3, 17, 18], available genetic maps remain at low to medium density, and are mainly based on a few hundred SSRs [19] and on a few hundred [20, 21] up to a few thousand [3, 18, 22] SNPs, usually developed through dedicated genotyping facilities. The development of larger resources is therefore required for mapping and genetic improvement purposes.

To complement the existing resources, our objective was to develop a comprehensive SNP resource in pea using genotyping by HiSeq sequencing of whole genome DNA and then to apply it for substantial genetic mapping. To our knowledge, this is the first report, in a species lacking a sequenced reference genome, of a whole genome genomic DNA sequencing strategy for high-throughput SNP discovery, genotyping (below called WGGBS) and genetic mapping. This novel approach was carried out at low sequence coverage, without prior genome complexity reduction and without sequence read assembly, on a RIL population segregating for *A.euteiches* resistance. The quality of the SNPs was then validated through genotyping using a benchmark technology [23]. This was made possible by optimizing SNP discovery tools that can work without data assembly or a reference genome [24], and developing appropriate tools to map very large numbers of SNPs from mapping populations comprising a few individuals [25].

## Results

### SNP discovery and selection of a subset of highly designable markers

To maximize the identification of relevant polymorphic SNPs, four genetically distant *P. sativum* genotypes were selected for genomic DNA preparation and HiSeq sequencing. These were the parental lines of the ‘Champagne’ x ‘Terese’ and ‘Baccara’ x ‘PI180693’ RIL mapping populations. Raw and pre-processed sequencing data analysis across the four samples showed low levels of contamination, and unexpectedly low levels of sequence repeats. In addition, considering that the nuclei were not isolated prior to DNA extraction, there were also rather low levels of organelle contaminants (with a higher level for the ‘Baccara’ sample, which was not etiolated). The final clean sequences represented 69 to 80 % of the raw sequence data depending on the genotype (Additional file 1: Table S1).

From a total of 1.32 billion cleaned reads from four *P. sativum* lines, the discoSnp tool identified 419,024 SNPs. A “post-discoSnp” filtering step, based on the availability of sequence data for all the four lines, homozygozity of each pea line, global sequence coverage and minor allele coverage, was used to remove putative “false heterozygous” and multilocus SNPs. Finally, 213,030 SNPs considered as robust for genotyping were selected. Most of them showed coverage between 6X and 14X (Additional file 2: Figure S1), which was consistent with the corresponding sequencing coverage for each line at approximately 7-fold the pea estimated genome size (Additional file 1: Table S1). As expected discoSnp filtering excluded SNPs with less than 5X coverage (Additional file 2: Figure S1). To optimize potential future GoldenGate® or KASP™ genotyping assay designs, only 131,850 SNPs were retained in two subsets of (i) 88,864 SNPs with a context sequence showing no other polymorphism at least 50 bp on either side of the SNP (considered as very highly designable) (ii) 42,986 SNPs with a context sequence showing no other polymorphism at least 50 bp on one side of the SNP and no other polymorphism at least 27 bp on the other side (considered as highly designable). Within the resulting 131,850 SNPs, polymorphic SNPs between pairs of parental lines ranged from 23,760 between the two spring sown field pea lines ‘Baccara’ and ‘Terese’ to over 97,000 between each of these two lines and the fodder pea genotype ‘Champagne’ (Table 1).

**Table 1:** Number of SNPs that were polymorphic between sequenced pairs of pea parental lines, from a subset of 131,850 highly or very highly designable SNPs

	Baccara	PI180693	Terese	Champagne
Baccara		88,851	23,760	97,705
PI180693			88,799	59,428
Terese				97,285
Champagne				

### SNP inferring on the ‘Baccara’ x ‘PI180693’ RIL mapping population

Forty-eight *P. sativum* RILs from the ‘Baccara’ x ‘PI180693’ mapping population were selected for genomic DNA extraction, Hiseq sequencing and genotyping.

Raw and pre-processed sequencing data analysis (Additional file 1: Table S1) across the 48 RILs was consistent with previous sequence analysis on the four parental lines, even with the twofold lower sequencing effort on the RILs (two lines per lane) than on the parental lines (one line per lane).

**Fig. 1:**
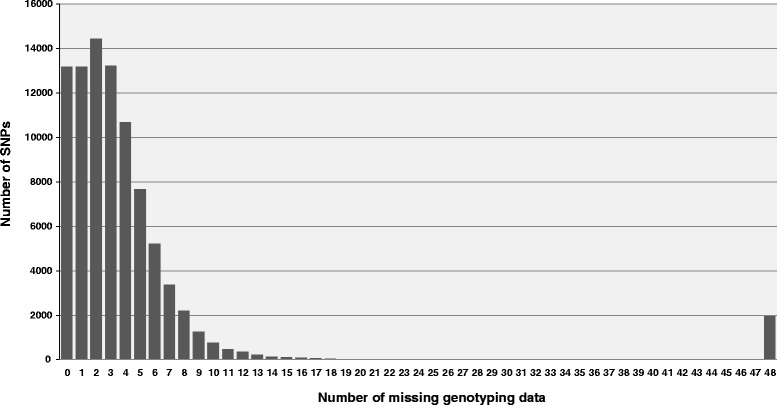
Frequency histogram of the number of missing data points in WGGBS of 48 RILs with the 88,851 reliable SNPs that are polymorphic between the ‘Baccara’ and ‘PI180693’ parentals (For example, 13,187, 13,186,and 14,452, were genotyped with 0, 1, or 2 missing data points, respectively among the 48 sequenced RILs)

A total of 88,851 SNPs (out of the 131,850 selected SNPs) were polymorphic between the ‘Baccara’ and PI180693’ parental lines. The kissreads module [24] of the discoSnp tool was then used to infer which of these SNPs were present in the 48 RILs. Most of these 88,851 SNPs showed coverage ranging between 3X and 7X (Additional file 2: Figure S1). This is consistent with the sequencing coverage for each of the 48 RILs of approximately 3.5-fold the estimated pea genome size (Additional file 1: Table S1). 13,187 SNPs were genotyped on all of the 48 sequenced RILs (Fig. 1). A total of 64,754 SNPs, which showed less than ten missing data points and less than 10 % of heterozygous data points among the 48 sequenced RILs, was retained.

A strict alignment of SNP context sequences with the Glint tool (http://lipm-bioinfo.toulouse.inra.fr/download/glint/) showed that only 482 SNPs are 100 % identical between the 35,455 SNP set developed by Duarte et al. [3], and the 88,851 ‘Baccara’ x ‘PI180693’ polymorphic SNP set generated in this study. Only 45 SNPs were found to be identical between the 604 ‘Baccara’ x ‘PI180693’ polymorphic SNP set genotyped in a GoldenGate® assay and mapped to the Duarte et al. [3] reference consensus map, and the 64,754 ‘Baccara’ x ‘PI180693’ polymorphic SNP set retained in this study from SNP inferring on the 48 RILs. Genotyping data obtained with both methods (GoldenGate® vs WGGBS) for the 45 common SNPs were identical for 39 SNPs, apart from a few missing data with each method and a few heterozygous loci identified with WGGBS but not GoldenGate®. The remaining six SNPs totally failed in the GoldenGate® assay but were successfully genotyped by direct sequencing (Additional file 3: Table S2).

### High density genetic maps

A first genetic ‘Baccara’ x ‘PI180693’ Duarte-derived map (below called BP-Duarte map), was constructed from the ‘Baccara’ x ‘PI180693’ polymorphic markers used in Hamon et al. [26] and Duarte et al. [3]. Positions of the resulting 914 mapped markers, covering 1073 cM, were generally colinear with those of the reference consensus map [3]. Linkage group (LG) lengths in cM were either similar or smaller in the BP-Duarte map than in the consensus map (Table 2).

**Table 2:** Comparative marker numbers, maps lengths and marker distributions per linkage group between the BP-WGGBS, the BP-Duarte and the consensus Duarte et al. [3] maps

	Number of markers			Length (cM)			Number of markers/cM			Number of gaps > 10 cM between two contiguous markers			Number of WGGBS developed SNPs newly mapped
	BP-WGGBS map	BP-Duarte map	Duarte et al. [3] reference consensus map	BP-WGGBS map	BP-Duarte map	Duarte et al. [3] reference consensus map	BP-WGGBS map	BP-Duarte map	Duarte et al. [3] reference consensus map	BP-WGGBS map	BP-Duarte map	Duarte et al. [3] reference consensus map	BP-WGGBS map
PsLGI	6163	93	235	118	140	147	52.2	0.7	1.6	1	1	0	6071
PsLGII	8995	102	260	171	173	218	52.7	0.6	1.2	1	1	1	8898
PsLGIII	12,868	162	339	181	189	203	70.9	0.9	1.7	0	0	0	12,706
PsLGIV	9785	133	270	133	146	169	73.6	0.9	1.6	0	0	0	9652
PsLGV	7634	120	265	139	134	156	55.3	0.9	1.7	0	0	0	7514
PsLGVI	8490	116	298	119	111	142	71.5	1	2.1	0	0	0	8373
PsLGVII	10,328	188	404	166	179	220	62.3	1	1.8	0	0	0	10,139
Whole	64,263	914	2071	1027	1073	1255	62.6	0.9	1.7	2	2	1	63,353

A second high-density ‘Baccara’ x ‘PI180693’ WGGBS-derived map (below referred to as the BP-WGGBS map), including 64,263 markers and covering 1027 cM, was constructed from genotyping data used for the BP-Duarte genetic map by adding the data for the selection of 64,754 SNPs “genotyped-by-sequencing” on the 48 ‘Baccara’ x ‘PI180693’ RILs (Table 2) to the previous matrix. This genetic map included 910 previously mapped markers and 63,353 (98.5 %) newly mapped genomic SNPs. SNP context sequences, genotyping data on parental lines and positions on the new BP-WGGBS of the 63,353 newly mapped genomic SNPs are described in Additional file 4: Table S3. Detailed mapping and polymorphism data in the 48 ‘Baccara’x‘PI180693’ RILs are included in Additional file 5: Table S4.

**Fig. 2:**
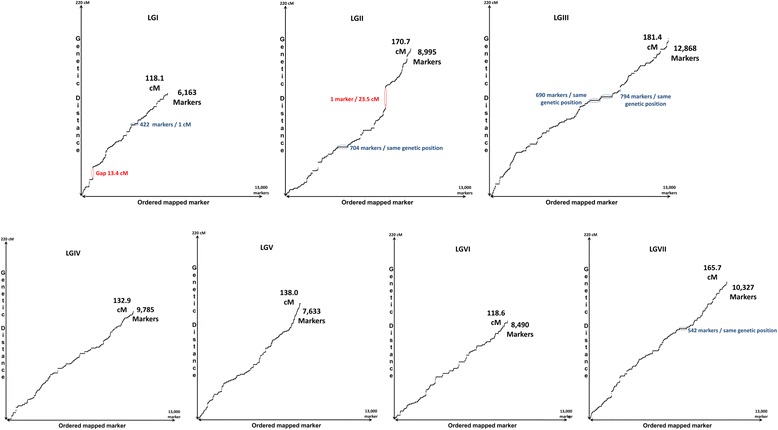
Dot-plot of marker distribution along the *P. sativum* linkage groups. A flatter curve indicates a region denser in markers. The red vertical ellipses indicate gaps without markers. The blue horizontal ellipses indicate hot-spots of markers at the same genetic position

This new BP-WGGBS map showed an average density of 62.6 markers per cM. Marker density was very high for all *P. sativum* LGs (PsLGs), and ranged from 52 markers/cM (PsLGI) to 74 markers/cM (PsLGIV) (Table 2). Overall, new SNP markers were usually densely and homogeneously distributed along the seven pea LGs, with a few notable exceptions: (i) two large areas remained without markers, i.e. two gaps larger than 10 cM between two contiguous markers (Table 2), located on PsLGI and PsLGII (Fig. 2), (ii) several spots showed 400 to 800 markers at the same genetic position, on PsLGI, PsLGII, PsLGIII (two very close spots), and PsLGVII. PsLGVI showed a “staircase” curve alternating marker dense and marker poor areas (Fig. 2).

Overall, 3.2 % of the newly developed SNPs showed segregation distortion (*P* < 0.01) among the 48 ‘Baccara’ x ‘PI180693’ derived RILs (Additional file 6: Figure S3). Most of the distorted markers clustered into genomic regions, mainly on PsLGs II (10.9 % of the mapped markers), III (5.4 %), IV (0.6 %), V (1.6 %) and VII (1.7 %). Markers in a defined cluster always distorted towards one of the parental lines (‘Baccara’ for clusters on PsLGs IV, V and VII, ‘PI180693’ for the cluster on PsLG III, and both parental lines on PsLGII for clusters on either side of the above described gap). PsLGI and PsLGVI showed negligible proportions of distorted markers.

Positions of the 914 markers common to the BP-Duarte and BP-WGGBS maps were colinear between the two maps, as well as with their published positions on the Duarte et al. consensus map [3] (Additional file 7: Figure S2). Except for a few local inversions, colinearity of these markers was maintained along the three maps. Map sizes were similar between the BP-Duarte and BP-WGGBS maps (respectively 1073 and 1027 cM), and significantly lower than the size of the Duarte et al. [3] reference consensus map from four populations (1255 cM). The number of mapped markers in the BP-WGGBS map was increased 31-fold and 70-fold compared to the consensus reference (comprising 2071 markers [3]) and to the BP-Duarte maps (Table 2).

### SNP inferring on other pea parental lines

SNP inferring on four supplementary sequenced parental lines showed that coverage of most of the 88,851 SNPs as expected ranged between 6X and 13X (Additional file 2: Figure S1). Among these the 63,353 newly mapped SNPs indicated a fair to high level of polymorphism between four other pairs of mapping population parents, ranging from 33 % to 64 % (Table 3, Additional file 4: Table S3). The SNP context sequence and polymorphism data for the eight *P. sativum* parents of the mapping populations are described in Additional file 4: Table S3.

**Table 3:** Percentage of polymorphic SNPs among the 63,353 newly developed SNPs mapped to the BP-WGGBS map, between five pairs of parental lines of pea mapping populations

*P. Sativum* LG	Nb of ‘WGGBS’ SNPs	% of polymorphic SNPs for 5 couples of mapping populations parents				
		Baccara & PI180693	Baccara & 552	Champagne & Terese	JI296 & DP	JI296 & FP
PsLGI	6071	100	35	64	34	37
PsLGII	8898	100	29	61	42	30
PsLGIII	12,706	100	40	67	40	29
PsLGIV	9652	100	38	66	36	34
PsLGV	7514	100	35	60	40	45
PsLGVI	8373	100	40	64	34	35
PsLGVII	10,139	100	43	63	32	26
Whole	63,353	100	38	64	37	33

### Selection and validation of a SNP sub-set in a KASP™ genotyping assay

Based on mapping positions within QTL confidence intervals for biotic stress resistance and polymorphism data, we selected 1000 SNPs out of the 63,353 mapped on the BP-WGGBS map. These were used for genotyping 1511 samples corresponding to 1438 different pea accessions (including the eight parental lines and the 48 RILs sequenced in this study), in a KASP™ 1,536-well plate format assay (see Methods).

Among these 1000 SNPs selected, 47 (5 %) failed due to missing or non-readable signals. Successful genotyping results were obtained with the other 953 SNPs (95 %), among which 949 (99.6 %) revealed the bi-allelic codominant polymorphism expected in the ‘Baccara’ x ‘PI180693’ RIL population. The remaining four markers (0.4 %) revealed a dominant polymorphism probably due to a failed probe design.

The 1,439,983 genotyping data points consisted of 47.3 % homozygous loci for alleles from ‘Baccara’, 48.1 % homozygous loci for alleles from ‘PI180693’, 1.3 % heterozygous loci, and 3.3 % unassigned data (failed, outside clusters, or null alleles). The entire polymorphism data set generated by the KASP™ genotyping assay in the eight sequenced pea parental lines and 4 F1s from crosses between pairs of these lines is described in Additional file 8: Table S5.

Forty-five thousand seven hundred forty-four comparisons were made between KASP™ and WGGBS genotyping data obtained with 953 SNPs on 48 RILs. Although there were higher levels of unassigned data generated with WGGBS than KASP™ genotyping, both methods gave very similar results for the 48 RILs genotypes (Additional file 9: Table S6). Conflicting data between the two methods remained below 1.5 % and included data: (i) where one method assigned a homozygote but the other a heterozygote (1 %), and which were mostly loci lying within regions showing residual heterozygosity and could therefore be re-assigned as heterozygotes; (ii) showing two different homozygotes assigned by either method (0.5 %), most of which (2/3) clustered in specific areas of a single RIL (BAP8_172) and may therefore correspond to a seed divergence and the remaining (1/3) that could be true genotyping errors from either method (Additional file 9: Table S6).

## Discussion

In this study, we discovered 419,024 genomic SNPs in whole genome sequences of four pea lines, but without prior genome reduction or sequence assembly. Among them, 213,030 appear robust for genotyping. For the first time in pea a WGGBS-derived map was produced. It contains 64,263 markers including 63,353 new genomic SNPs added to 910 other markers that allowed this new high-density genetic map to be aligned to a previously published reference consensus map (Duarte et al., [3]).

### Whole genome DNA sequencing: a strategy without genome reduction for high density development of markers in an orphan species

SNP development strategies usually call for a genome reduction step, either focusing on the expressed fraction of the genome, using tags or methylation at restriction sites, or capture, in order to produce sequencing data with a sufficient coverage to avoid false polymorphisms. Although the pea genome is large (4.3 Gb) and thought to contain a large proportion of repeated sequences, we chose not to go through this genome reduction step and to work on direct whole genome DNA sequencing, associated with reduced coverage thresholds to validate the SNPs. This strategy generated more than ten billion 100 bp high quality reads from eight pea lines and 48 RILs, with a high level of polymorphism validation in other sequenced parental lines or in derived RILs. Results of the cleaning process were consistent for all lines, except ‘Baccara’ which was not etiolated prior to tissue sampling. The unexpectedly low level of repeated sequences (10 to 15 %) could be because the mapping parameters in the cleaning method were very stringent or some pea genome repeated sequences are under-represented in the REPbase and Genbank databases. Regardless, the remaining repeated sequences were taken into consideration during SNP discovery with the discoSnp tool and post discoSnp filtering processes. Thus we did not observe high SNP coverage that could have corresponded to organelle or repeated sequence regions (Additional file 2: Figure S1). The upcoming availability of the whole pea genome sequence will give further insights into the quantity and nature of its repeated sequences [14].

### SNP calling without data assembly and without a reference genome

For a genomic SNP discovery approach, a tool appropriate for a large genome without reference sequence and a large amount of data was a prerequisite. We therefore used discoSnp software [24] that filled the specifications for SNP discovery from non-assembled reads. This tool, designed for calling SNPs from any number of input read sets, avoids the assembly and mapping processes, and therefore needed very limited time and memory to process ten billion reads. SNP discovery on the four parental lines only needed 1 CPU, 4 GB RAM and 48 h. SNP inferring with the kissreads module of discoSnp on the 48 RILs used 24 CPU, 4 GB RAM and 20 h. discoSnp outputs pairs of sequences in a fasta file. Sequences in the pair differ by a unique isolated nucleotide polymorphism, ie without any other polymorphisms in the context sequence on either side of the SNP. Although this is a strong limitation to the comprehensive detection of all possible SNPs, it is perfect for specifically selecting SNPs that can be directly used to design SNP-based assays for genotyping such as Illumina GoldenGate®, LGC Kasp™ or Affymetrix Axiom®.

The header of the discoSnp output sequences contains information about the read quality and coverage of each of the two alleles in each of the input read sets. Thus we could apply filters specifically adjusted to our biological parameters. One filter removed false heterozygotes (previously undetected) due to low coverage and low depth of whole genome sequencing data. Another filter, based on the coverage of the minor allele for SNPs detected on four pea lines, was used to remove questionable SNPs for complexity, multilocus status, copy number variations, or potential location in genome repeats. After this stringent filtering step, around 50 % of the 419,024 SNPs for all four pea parental lines were retained. This is a much higher figure than currently reported for GBS strategies which usually result in high levels of missing sequence data and a large percentage of uncalled genotypes [27, 28]. Indeed, whole genome sequencing (WGS) inherently provides very good quality sequence data that are more comprehensive than GBS. However the combined use of a tool such as discoSnp and a “post-discoSnp” specific filtering strategy were needed for efficient SNP discovery process. Furthermore, the level of missing data after sequencing 48 RILs was also very low, with 64,754 (among 88,851) SNPs showing less than 20 % missing sequence data (Fig. 1). To our knowledge this is the first study conducted using discoSnp on a real data set of reads from HiSeq sequencing. It had been previously used on a much smaller 454 read set after reduction of the *Ixodes ricinus* genome, to detect “ready to genotype” SNPs [29].

Only 482 of the 88,851 newly discovered polymorphic SNPs (between ‘Baccara’ and ‘PI180693’) were in common with the 35,455 gene-based SNPs developed by Duarte et al. [3]. This may be due to (i) relatively lower coverage and lower sequencing depth provided in this study, (ii) the specific detection of only isolated SNPs by discoSnp. Recently, Alves Carvalho et al. compared three SNP calling methods, including discoSnp, on the same set of sequence data. Each detected the same numbers of robust SNPs, but only a quarter of the SNPs were in common in the three sets and only half between two sets [30].

### Validation of the WGGBS genotyping strategy using a SNP KASP™ genotyping assay

Despite the rather low average sequencing coverage in SNP discovery (around 7x) and in further SNP inferring (around 3.5x), several biological and technical observations confirmed the robustness of our strategy and showed its full efficiency in revealing reliable and designable SNPs. The polymorphism levels revealed between pairs of lines were consistent with previously described genetic distances between these lines [3]. Indeed, polymorphism levels were lower for ‘Baccara’/‘Terese’ than for ‘Baccara’/‘Champagne’, ‘Champagne’/‘Terese’ or ‘Baccara’/‘PI180693’. ‘Baccara’ and ‘Terese’ belong to the spring field pea group, ‘Champagne’ to the winter fodder pea group, and ‘PI180693’ to the garden pea group. Similarly, genotyping data for the 45 SNPs in common with the 604 SNP set genotyped in a GoldenGate® assay and mapped by Duarte et al. were entirely consistent with the 64,754 genomic SNPs generated and genotyped-by-sequencing in this study (Additional file 3: Table S2). Furthermore, almost all of the 88,851 SNPs polymorphic between ‘Baccara’ and ‘PI180693’ were successfully genotyped on the RIL population (Fig. 1). Genotyping of 1438 different pea accessions with a subset of 1000 SNPs (1.4 % of the overall newly generated SNP resource) using the KASP™ technology also revealed a very high rate of 953 true and reliable SNPs (95 %). Thus, the chosen strategy of low average sequencing coverage (around 7x) followed by discoSnp-based SNPs discovery was fully validated.

Finally, genotyping data obtained with both KASP™ and WGGBS methods for the 953 SNPs on the 48 RILs were very similar. The level of missing data (almost twofold higher with WGGBS than with KASP™), as well as the 1.5 % inconsistent data between methods, suggests that WGGBS is less robust in heterozygote discrimination, probably due to the low sequencing depth. However, WGGBS was highly efficient and robust in revealing homozygous genotypes. Thus we confirmed that the low average sequencing coverage (around 3.5X) coupled with the use of the kissreads module of discoSnp inferred robust and reliable genotyping data.

### High-density genetic map construction from a *P. sativum* RIL population

Most developments in genotyping techniques in the last ten years have been clearly associated with multiplexing large numbers of markers (up to millions for example in Illumina or Affymetrix genotyping arrays) for each plant analyzed. WGGBS approaches tend to also provide huge quantities of SNP allele information for each plant. This leads to new challenges when building linkage maps. The main problem is that the number of plants in the mapping population is always far too small for a robust ordering of all the polymorphic markers. Thus, two distinct objectives have to be considered to produce: (i) a robust map with the largest possible number of markers (but still moderate due to limited population size) for which the order is statistically supported for a given LOD threshold; and (ii) a “complete map” in which all polymorphic markers are given a position, but without the order between markers being statistically supported (this is the principle of “bin-mapping” [31]). Another technical problem is the need for most mapping software to load the whole set of marker segregation data in memory, which makes it impossible to work with data sets beyond a certain size. The pipeline used in this study, developed in response to these different challenges, makes it possible to build both framework maps with orders supported by a LOD threshold and complete maps saturating the genomic space with all polymorphic markers [25]. Thus the map is suitable for QTL detection, GWAS and genomic selection approaches. In addition, marker data are loaded sequentially in small batches, so there is no limit to the number of markers, regardless of computer memory size. This sequential approach needs a much shorter computation time compared to the direct use of the annealing command of the CAR_H_^T^A GENE tool: about 10 h were needed to map 64,000 SNPs on the seven PsLGs, whereas annealing commands in CAR_H_^T^A GENE would probably have taken months or years. We are currently working on parallelizing the code for multicore computers to further reduce this computation time.

This pipeline allowed us to generate the first high-density pea genetic map mainly based on SNPs developed and genotyped by sequencing whole genome genomic DNA from a RIL population. The map size obtained was significantly smaller than those observed in previous reference composite maps [3, 18, 32], and similar to the sizes of recently reported individual maps [22] and the BP-Duarte individual map (this study), but with a more than 70-fold increase in marker density. A very high level of colinearity was observed for the markers that were in common with other *P. sativum* composite [3, 18, 32] or individual ([22], BP-Duarte-derived) maps. Thus the newly developed BP-WGGBS map is a useful tool for future studies focusing on variation in a given genomic region in pea.

A 3.2 % segregation distortion (*P* < 0.01) was observed for the newly developed SNPs among the 48 ‘Baccara’x‘PI180693’ derived RILs. This figure is very close to the 3 % identified for 224 markers in the ‘Baccara’/‘PI180693’/‘552’ consensus map [33] and the 4.5 % identified for SNPs in the ‘Baccara’ x ‘PI180693’ individual population data [3]. However it is much lower than the segregation distortion observed in other individual pea populations where large numbers of SNPs were genotyped and mapped [3, 17, 18]. The much higher marker number involved and the high resolution of the BP-WGGBS map allowed regions of segregation distortion to be identified in this population towards one or the other parental line, probably carrying detrimental lethal or sublethal alleles. Although the two parental lines are actually genetically distant [3], sterility barriers were not noted in the RIL production and fixation process. It is possible however that gametic or zygotic selection did take place and the issue of whether floral development or fertility genes lie in these regions still needs to be addressed. Genotypic data for distorted markers was not specifically considered during the mapping process. It is possible therefore that some marker order inversions occur in the distorted regions. However, considering that all the markers are not distorted in a specific region and the WGGBS-derived map aligns well with other maps (Additional file 7: Figure S2), such inversions are probably localized and infrequent. Information on marker distortion combined with future knowledge of localized recombination hot and cold spots, is of vital interest for some of the genomic regions that control traits of interest in this population. These are currently transferred through back-cross assisted breeding from ‘Baccara’ x ‘PI180693’ RILs to field pea spring and winter sown elite genetic backgrounds [34]. Interestingly a 23 cM gap on PsLGII separated two regions where markers showed distortions towards either parental line. Although a single marker ensures the link between these two regions, it is validated by other individual ‘Baccara’ x ‘PI180693’ maps (Duarte derived map from this study) or consensus maps where the gap was also observed [19, 33] or not [3, 18]. It is likely that in this region showing no recombination a chromosomal rearrangement occurred in the ‘Baccara’ x ‘PI180693’ population.

By choosing to sequence whole genomic DNA, we developed a large original resource of genomic SNP markers corresponding to genic and extra-genic sequences distributed all along the pea genome. Like genomic SSRs, but unlike cDNA-derived SNPs, it can be considered as neutral regarding evolutionary selection pressure. This was confirmed by the dense and homogeneous distribution of newly developed SNP markers along the seven pea LGs. A remaining 23 cM gap on PsLGII (Fig. 2) corresponds to a misassembly of two large blocks more than 30 cM apart, or to a 25 cM region without polymorphic markers between ‘Baccara’ and ‘PI180693’ comprising three gaps of 13.1, 4.6, 4.4 cM on two previous reference consensus maps [3, 19]. Interestingly no such gap was observed on the Sindhu et al. consensus map [18] that did not include the ‘Baccara’ x ‘PI180693’ RIL population. Furthermore, the atypical distribution of markers along the PsLGVI with a “staircase” curve alternating marker dense and marker poor areas (Fig. 2), is consistent with what we know of the complex synteny with *M.truncatula* pseudo chromosomes 2 & 6 [3].

### A comprehensive tool for academic research and breeding in pea: the case of an *A. euteiches* resistance QTL

**Fig. 3:**
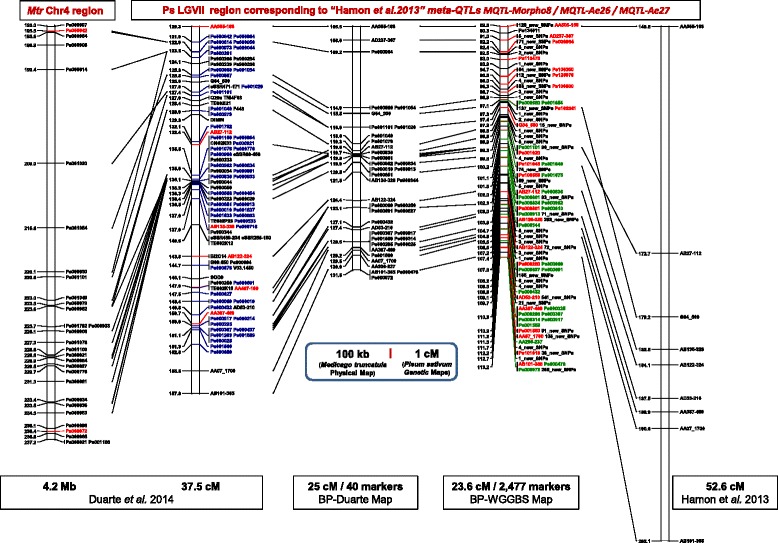
Marker densification in a MetaQTL region controlling partial resistance to *A.euteiches* between the SSR AA505 and AB101 reference markers on PsLGVII. The left hand side shows this region on the Duarte et al. [3] consensus map and its projection on *M. truncatula* pseudochromosome 4 (from Duarte et al. [3] - Additional file 5: Table S4). The center shows the same region on the two individual BP-Duarte and BP-WGGBS maps, covering respectively 25 cM and 23.6 cM. The right hand side shows the same region, detailed in Hamon et al. [26], covering 52.6 cM and corresponding to three MetaQTLs Morpho8, Ae26 and Ae27 (from Hamon et al. [26] - Additional files 9: Table S6 and Additional file 10: Table S7)

Sequencing and mapping a RIL population segregating for partial resistance to *A. euteiches* should lead to breakthroughs in the study of QTLs involved in *A.euteiches* resistance [26, 33]. There is now the potential for densification, fine mapping and candidate gene identification within the QTL confidence intervals. In addition, alignment of the BP-WGGBS map with the Duarte et al. [3] consensus map [3], anchors some of the markers to the genome of the model species *M.truncatula,* and opens the way for large-scale syntenic studies. The “metaQTL” area described by Hamon et al. [26] on a PsLGVII region corresponding to flowering MetaQTL Morpho8, and *A. euteiches* resistance MetaQTLs Ae26 and Ae27 is for instance defined between the SSR markers AA505 and AB101. This region originally covered 52.6 cM and included eight markers. Now it covers a much shorter genetic distance (23.6 cM) and includes 2,477 markers on the ‘BP-WGGBS’ map. The upper section of this region (the distal part has no clearly defined syntenic block) can also be linked to a 4.2 Mb region of *M.truncatula* chromosome 4 using around 30 gene-derived bridge markers (Fig. 3). Mapping, alignment and redetection of *A.euteiches* resistance QTL from Hamon et al. [26] are currently in progress [26].

The alignment of this new BP-WGGBS map to the consensus reference map [3] from four RIL populations including ‘Baccara’ x ‘PI180693’ , will also facilitate the use of the SNP resource to fine map genomic regions and QTLs associated with traits of interest identified from other RIL populations where maps with common markers are available [22, 32, 35]. As shown by the level of polymorphism observed in five pairs of mapping population parents (Table 3) from sequencing only four pea lines (Table 3), any pea line can be rapidly and cheaply sequenced to obtain polymorphism information for the newly developed and mapped SNPs. For example, the PsLGVI region bordered by Ps001502 and FVE markers including a major QTL for winter frost damage [22] and syntenic with a 6.97 Mb region on the MtrChr8 [3] potentially includes 1,669 markers from the BP-WGGBS map.

## Conclusion

In this study, a major genomic SNP marker resource was generated. It will be extremely valuable in future research of the genetic control of traits of interest, and breeding for the introgression and management of these traits into cultivated gene pools. The results show that it is now possible to generate and map reliable SNP markers in a plant species with a large unsequenced genome. The novelty of our approach included using whole sequencing without genome reduction, direct SNP discovery excluding sequence assembly, and a sequential mapping approach to handle tens of thousands of markers. The entire set can be used in single gene, association, or linkage mapping studies to capture new QTLs and refine QTL localizations. This can be done in any pea genetic background since mapping alignments are provided and the markers show high levels of polymorphism in alien backgrounds. The resource can also be screened in linkage studies (fine mapping, introgression) aimed at specific regions when generating large segregating populations. Finally, we generated a large choice of polymorphic markers internal to and bordering QTL confidence intervals. These provide an unprecedented tool for Marker Assisted Selection.

## Methods

### Plant material and tissue sampling

A first set of plant material consisting of eight *P. sativum* genotypes was selected for (i) being parental lines of mapping populations (ii) including sources of partial resistance or tolerance to biotic and abiotic stresses and (iii) examining polymorphisms within or between *P. sativum* cultivated types, i.e. fodder, field (winter sown and spring sown), and garden pea types (Additional file 10: Table S7).

A second set consisted of 48 F8 Recombinant Inbred Lines (RILs) selected from a 178 RIL mapping population developed by Single Seed Descent from the cross between the cultivar ‘Baccara’ and the ecotype ‘PI180693’ [26, 33]. The 48 RILs were part of a 90 RIL set of the population that was used to establish a previous reference composite map [3]. The 48 RILs were sampled using MapPop 1.0 software [36] which was designed to select informative individuals optimizing the distribution of recombination points all over the genome. The “sampleexp” command was used to select the RIL set minimizing the expected Average Bin Length (eABL), i.e. the distance between two recombination points, using ‘Baccara’ x ‘PI180693’ genotyping and genetic map data from Hamon et al. [33] as the input file.

The 56 *P. sativum* genotypes were grown in a climate-controlled chamber (16 h photoperiod, temperature 15 °C night/20 °C day, 60 % minimal hygrometry). At least five plants per genotype were collected 15 days after sowing including a final etiolation period of five days (except for the Baccara sample). Tissues were flash frozen in liquid nitrogen and stored at −80 °C until further use.

### DNA extraction

Genomic DNA was extracted from leaf tissue using a CTAB method as described by Rogers and Bendich [37]. The quality and quantity of extracted DNA was evaluated using a NanoDrop 8000 spectrophotometer (Thermo Fisher Scientific) and the Quant-iTTM dsDNA Assay Kit (Invitrogen). An estimated quantity of 3 μg of total genomic DNA was used to prepare each library, in a volume of 130 μL of ultra-purified water.

### Library preparation and sequencing

DNA libraries were prepared using the Illumina TruSeq DNA protocol following manufacturer’s guidelines. Briefly, DNA was fragmented using a Covaris M220 sonicator. DNA was then end repaired and A-tailed, followed by the ligation of adapters and 12 cycles of PCR. Library profiles were controlled on a BioAnalyzer High Sensitivity chip. Quantities of usable material for each of the libraries were estimated by qPCR (KAPA Library Quantification Kit–Illumina Genome Analyzer-SYBR Fast Universal) and then normalized and pooled. The quality of the pools was then checked using qPCR and immediately followed by sequencing on the HiSeq2000 platform (Plateforme Genomique - Genopole Toulouse Midi-Pyrenees, France), using TruSeq PE Cluster Kit v3 (2 x 100 pb) and TruSeq SBS Kit v3. Eight lanes, each generating on average 36 Gb of sequences (360 M reads x 100 pb length) were necessary to sequence the set of eight lines. Twenty-four lanes, each generating on average 38 Gb of sequence (190 M reads x 100 pb length), were necessary to sequence the 48 RILs. Raw data were produced as sff files.

### Sequence cleaning

Raw sequence data were processed with a dedicated pipeline enchaining different cleaning steps: after excluding contaminants, Flexbar [38] was used to remove sequence adapters. Strict read mapping was then performed with BWA [39] at first against RepBase [40], Medicago repeat library from TIGR Plant Repeats (ftp://ftp.plantbiology.msu.edu/pub/data/TIGR_Plant_Repeats/TIGR_Medicago_Repeats.v2), and available pea repeat sequences from Genbank. BWA was used subsequently to remove mitochondrial (from *M.truncatula*, *A.thaliana* and *Glycine max* NCBI) and chloroplastic (from pea NCBI RefSeq NC_014057.1) contamination. Low complexity sequences were finally removed using a custom perl script.

### SNP discovery on four pea lines without data assembly

The discoSnp tool [24] calls SNPs from one or several read sets without using a reference genome or any other source of information. DiscoSnp aims to predict isolated SNPs (well suited for being easily amplified by PCR), k nucleotides apart from any other polymorphism source, with k being the main parameter. A micro assembly approach generates a fasta file containing each identified SNP with the contig it belongs to, represented by a pair of sequences which differ only at the polymorphic site. Each sequence comment provides information on average read coverage and average read quality per input read set. We applied the discoSnp tool to cleaned reads from four pea lines (‘Baccara’ , ‘PI180693’ , ‘Champagne’ and ‘Terese’), with “k-mer = 27” as the input parameter, which was empirically shown to maximize the number of predictions in our conditions [24]. The kissreads module of the discoSnp tool provides the coverage for each SNP on the reads for each RIL. A dedicated perl script then enchained several filters to retain only the most reliable SNPs based on available sequence data for the four pea lines, strict homozygozity of each pea accession, a minimum 5x coverage on at least one pea accession, and a coverage of the minor allele at least half that of the major allele.

### SNP inference and whole genome genotyping by sequencing

Robust SNPs (highly or very highly designable for genotyping assay designs) polymorphic between the ‘Baccara’ and ‘PI180693’ parental lines were inferred using the kissreads module of the discoSnp tool [24] on cleaned reads generated by sequencing the 48 ‘Baccara’ x ‘PI180693’ RILs. Kissreads provides the coverage for each SNP on reads for each RIL. Considering that less sequence data was generated for RILs than the parental lines, and that a SNP which is inferred on already identified SNPs is robust, a 3x coverage threshold was applied to each RIL read set to select reliable data.

To identify potential polymorphisms in a larger set of parental lines, SNPs were inferred in the same SNP set as used for the 48 RILs using the kissreads module. It was applied to the cleaned reads generated by sequencing four additional parental lines of mapping populations (‘552’, ‘JI296’, ‘DP’ and ‘FP’). A 5x coverage threshold was set in order to select reliable data.

For each SNP, the allele was coded “A” when identical to the ‘Baccara’ parent, or “B” when identical to the ‘PI180693’ parent.

### Construction of BP-Duarte and BP-WGGBS genetic maps

To first build a robust individual ‘Baccara’ x ‘PI180693’ map, we used genotyping data from the Duarte et al. [3] reference consensus map. All the markers that were polymorphic for the 90 ‘Baccara’ x ‘PI180693’ RILs were used for the consensus map. We also used genotyping data obtained from mainly SSR [26] and SNP [3] markers on the entire ‘Baccara’ x ‘PI180693’ 178 RIL population. The final genotyping data matrix comprised a total of 928 markers, including 295 markers with data on 178 RILs and 633 markers with data on a sub-set of 90 RILs. The 1:1 allelic segregation ratio for each marker within the RIL population was verified using a Chi-square test (P > 0.01 and P > 0.001). Genetic linkage analyses were performed using the “group” command of CAR_H_^T^A GENE software [41], with a minimum LOD score threshold of 3.0 and a recombination frequency <0.3. Marker order was refined using the “annealing 100 100 0.1 0.9” command of CAR_H_^T^A GENE. The resulting map was called BP-Duarte.

To build the new map including the WGGBS data, we added to the previous matrix the “genotyping by sequencing” data obtained in this study on 48 ‘Baccara’ x ‘PI180693’ RILs. The 1:1 allelic segregation ratio at each marker was verified and the LGs established as for the previous genetic map. Then each LG was constructed individually as described earlier [25] using CAR_H_^T^A GENE called from custom R scripts (http://www.r-project.org/foundation). In the first step, statistically robust scaffold maps were constructed by elongating the map from one seed marker in both directions with the most strongly linked marker in the data set, located at a distance greater than 5 cM. This procedure ensured an extremely robust order of regularly-spaced markers. To avoid potential detrimental consequences due to the choice of the seed marker, for instance on incomplete map coverage, 10 independent replicates were performed using ten randomly drawn seed markers, and a consensus scaffold was built by merging all the ten scaffolds. In a second step, marker density of the consensus scaffolds was increased to produce framework maps containing as many markers as possible, while keeping a LOD score >3.0 for the robustness of marker orders. Finally, the complete map was obtained by individual placement of additional markers on the framework map using bin mapping [31]. Markers with a minimum allele frequency less than 6 % and markers with more than 78 % of missing data were not included on the scaffold and framework maps, but they were included in the final placement step, since this could not influence the placement of other markers. The resulting map was called BP-WGGBS.

For both the BP-Duarte and BP-WGGBS genetic maps, the Haldane function was used to calculate cM distances between markers [42] and MapChart 2.2 was used to draw the maps [43].

### Selection and validation of a 1000 SNP subset in a 1536-well plate KASP™ genotyping assay

A subset of 1000 SNPs was chosen among the mapped SNPs on the BP-WGGBS genetic map to design a 1536 well plate KASP™ [23] assay. SNP selection was based on: (i) an even distribution of markers in genomic regions containing QTL of interest for resistance to various biotic stresses; (ii) one single SNP per genetic position whatever the number of fully linked markers at this position; (iii) highest polymorphism levels between pairs of parental lines of mapping populations among the eight sequenced lines. The final selected SNP subset covered around 500 cM of the 1027 cM BP-WGGBS genetic map and can be considered as a quality-unbiased sampling of the whole SNP resource generated. SNP designability on KASP™ technology showed a 99 % success rate (LGC genomics service lab, UK). For each assay, at least 15 μg of genomic DNA, i.e. 16 96-well plates each containing 100 μL of samples were provided to LGC Genomics service lab, UK (http://www.lgcgenomics.com) for sample normalization and genotyping using KASP™ technology [23]. A total of 1511 DNA samples (corresponding to 1438 different pea accessions, including the eight parental lines and the 48 RILs sequenced in this study, and a large number of other RILs and accessions), were genotyped. Automatic allele calling for each locus was carried out using Klustercaller software [23]. The homozygous and heterozygous clusters were checked visually and were manually edited when necessary. Technical replicates and signal intensities were verified and only the most reliable calls were retained.

## Availability of supporting data

SNP markers were directly identified using the discoSnp tool without sequence assembly. The entire set of cleaned reads that was generated in this project has been deposited in FASTQ format at ENA/PRJEB9689 (http://www.ebi.ac.uk/ena/data/view/PRJEB9689) under the accessions ERS762237-ERS762244 for the eight sequenced pea lines and ERS762253-ERS762300 for the 48 sequenced pea RILs.

## Additional files

Additional file 1: Table S1. Statistics on raw, cleaned and processing of sequencing data across the eight parental lines and 48 ‘Baccara’ x’PI180693’ pea RILs sequenced. (PDF 223 kb) Additional file 2: Figure S1. Frequency histograms of SNP coverage. (A) corresponds to 419,024 SNPs identified by discoSnp on four pea lines, (B) to 213,030 SNPs selected after post discoSnp filtering on four pea lines, (C) to a selection of 88,851 polymorphic SNPs on 48 pea RILs, (D) to a selection of 88,851 polymorphic SNPs on eight pea lines. (PDF 267 kb) Additional file 3:Table S2. Comparative genotyping of the 48 ‘Baccara’ x ‘PI180693’ RILs using 45 SNPs genotyped both through a GoldenGate**®** Assay (Duarte et al., 2014) and direct Whole Genome Genotyping by Sequencing (this study). SNP codes Ps0xxxxx and Ps0xxxxx_SNP_path_xxxxxx correspond to the GoldenGate**®** assay and to the direct sequencing assay, respectively. “A” corresponds to a ‘Baccara’ homozygous parental genotype, “B” to a ‘PI180693’ homozygous parental genotype, “H” to an heterozygous genotype, “-” indicates missing data. (PDF 332 kb) Additional file 4: Table S3. Context sequences, genetic positions on pea linkage groups and genotyping data for eight *P. sativum* parental lines of mapping populations with a set of 63,353 newly developed SNP markers mapped to the seven linkage groups of the BP-WGGBS map. (XLSX 8728 kb) Additional file 5: Table S4. Mapping data and polymorphism in the 48 ‘Baccara’ x ‘PI180693’ RILs of a set of 64,263 SNP markers mapped to the seven linkage groups of the BP-WGGBS map. (XLSX 17036 kb) Additional file 6: Figure S3. Localisation and proportions of non-distorted (P > 0.01) and distorted (*P* < 0.01) markers along the linkage groups of the pea BP-WGGBS map. Red and blue ellipses indicate distorsions towards the ‘Baccara’ and ‘PI180693’ parental alleles, respectively. X axis is distance in cM, Y axis is Chi-square probability. (PDF 1114 kb) Additional file 7: Figure S2. Colinearity of 914 markers common to the BP-WGGBS (left) and BP-Duarte (right) maps with the Duarte et al. (2014) reference consensus map (middle). (PDF 337 kb) Additional file 8: Table S5. Polymorphism data in the 8 sequenced pea parental lines and 4 F1 selected from a set of 953 SNP genotyped in LGC Kasp™ assay methods on 1438 different pea accessions. “A” corresponds to ‘Baccara’ homozygous parental genotype, “B” to ‘PI180693’ homozygous parental genotype, “H” to an heterozygous genotype, “-” indicates uncalled data. For each pea line, if available, different seed lots were genotyped (seed-DNA lot numbers in parenthesis). (XLSX 115 kb) Additional file 9: Table S6. Comparing polymorphism data in the 48 ‘Baccara’ x ‘PI180693’ RILs from a set of 953 SNP genotyped both in WGGBS and LGC Kasp™ assay methods, ordered according to their genetic position on the BP-WGGBS map. “A” corresponds to ‘Baccara’ homozygous parental genotype, “B” to ‘PI180693’ homozygous parental genotype, “H” to an heterozygous genotype, “-” indicates uncalled data. (XLSX 439 kb) Additional file 10: Table S7. Parental accessions with partial resistance (PR), tolerance (TOL) or susceptibility (S) to stresses, used for sequencing and/or genotyping. (PDF 347 kb)

## Abbreviations

BP-: ‘Baccara’ x ‘PI180693’
cDNA: complementary deoxyribonucleic acid
cM: centimorgan
CPU: central processing unit
CTAB: cetyltrimethylammonium bromide
DNA: deoxyribonucleic acid
Gb: gigabase
GB: gigabyte
GBS: genotyping-by-sequencing (method)
GWAS: genome-wide association study
KASP: kompetitive allele specific PCR
LG: linkage group
LOD: logarithm (base 10) of odds
NGS: next-generation sequencing
PCR: polymerase chain reaction
PsLG: *pisum sativum* linkage group
QTL: quantitative trait locus
RAD: restriction site associated DNA (markers)
RAD-Seq: restriction site associated DNA sequencing
RAM: random-access memory
RIL: recombinant Inbred Line
RNA: ribonucleic acid
SNP: single-nucleotide polymorphism
SSR: simple sequence repeats (markers)
WGGBS: whole genome genotyping by sequencing
WGS: whole genome sequencing
