# Supplementary material for: SNP discovery and genetic mapping using genotyping by sequencing of whole genome genomic DNA from a pea RIL population
Source: BMC Genomics. 2016 Feb 18;17:121. doi: 10.1186/s12864-016-2447-2 (PMC4758021; doi:10.1186/s12864-016-2447-2)
Supplement: Additional file 1: Table S1. — Statistics on raw, cleaned and processing of sequencing data across the eight parental lines and 48 ‘Baccara’ x’PI180693’ pea RILs sequenced. (PDF 223 kb) [file 12864_2016_2447_MOESM1_ESM.pdf]

Lines	Raw Data		Cleaned Data			Processing details				
	Nb.raw reads	Nb. Raw bases	Nb. cleaned reads	Nb. cleaned bases	% clean datas	Contam.	Flexbar	RepBase	ChloroMito	LowComp.
**Baccara**	406,540,758	40,654,075,800	280,842,653	28,084,265,300	69.08%	0.15%	0.35%	4.34%	20.79%	5.29%
**Champagne**	507,344,040	50,734,404,000	407,152,922	40,715,292,200	80.25%	0.17%	0.23%	5.04%	8.68%	5.63%
**PI180693**	397,209,864	39,720,986,400	316,433,849	31,643,384,900	79.66%	0.10%	0.28%	5.04%	9.39%	5.52%
**Terese**	400,845,472	40,084,547,200	319,546,103	31,954,610,300	79.72%	0.15%	0.28%	5.10%	9.23%	5.53%
**DP**	403,480,176	40,348,017,600	328,979,812	32,897,981,200	81.54%	0.90%	0.52%	5.01%	4.21%	7.82%
**FP**	358,449,446	35,844,944,600	301,504,061	30,150,406,100	84.11%	0.51%	0.65%	4.91%	4.54%	5.28%
**JI296**	385,808,764	38,580,876,400	313,384,634	31,338,463,400	81.23%	0.55%	0.64%	4.78%	4.67%	8.13%
**552**	379,324,340	37,932,434,000	307,673,430	30,767,343,000	81.11%	0.68%	0.19%	4.86%	5.21%	7.94%
**BAP8-004**	216,369,986	21,636,998,600	166,989,709	16,698,970,900	77.18%	0.17%	0.15%	5.04%	12.41%	5.06%
**BAP8-006**	164,477,702	16,447,770,200	131,659,794	13,165,979,400	80.05%	0.19%	0.15%	5.06%	9.35%	5.20%
**BAP8-018**	170,059,238	17,005,923,800	127,902,506	12,790,250,600	75.21%	0.20%	0.14%	4.86%	14.77%	4.82%
**BAP8-024**	158,844,376	15,884,437,600	126,992,492	12,699,249,200	79.95%	0.23%	0.14%	4.92%	9.48%	5.29%
**BAP8-029**	183,855,614	18,385,561,400	147,679,892	14,767,989,200	80.32%	0.10%	0.14%	4.98%	9.25%	5.21%
**BAP8-037**	249,509,326	24,950,932,600	199,052,678	19,905,267,800	79.78%	0.10%	0.15%	5.20%	10.08%	4.70%
**BAP8-043**	236,017,008	23,601,700,800	193,593,790	19,359,379,000	82.03%	0.09%	0.15%	5.23%	7.63%	4.88%
**BAP8-047**	157,869,112	15,786,911,200	124,873,035	12,487,303,500	79.10%	0.08%	0.14%	4.95%	10.65%	5.07%
**BAP8-051**	196,313,900	19,631,390,000	159,886,340	15,988,634,000	81.44%	0.13%	0.21%	4.97%	7.34%	5.90%
**BAP8-056**	203,551,320	20,355,132,000	160,110,648	16,011,064,800	78.66%	0.17%	0.18%	4.77%	10.60%	5.62%
**BAP8-057**	204,871,626	20,487,162,600	169,472,602	16,947,260,200	82.72%	0.18%	0.16%	5.03%	5.90%	6.01%
**BAP8-060**	187,978,064	18,797,806,400	152,057,628	15,205,762,800	80.89%	0.21%	0.14%	4.87%	7.60%	6.29%
**BAP8-066**	196,793,484	19,679,348,400	162,356,746	16,235,674,600	82.50%	0.17%	0.17%	5.10%	6.24%	5.81%
**BAP8-070**	174,838,562	17,483,856,200	136,477,750	13,647,775,000	78.06%	0.16%	0.21%	4.86%	10.83%	5.87%
**BAP8-082**	238,914,618	23,891,461,800	188,761,171	18,876,117,100	79.01%	0.15%	0.21%	4.83%	9.92%	5.88%
**BAP8-090**	163,000,228	16,300,022,800	129,767,439	12,976,743,900	79.61%	0.12%	0.19%	4.63%	9.31%	6.13%
**BAP8-094**	159,002,174	15,900,217,400	126,492,445	12,649,244,500	79.55%	0.18%	0.13%	4.94%	9.65%	5.55%
**BAP8-099**	176,491,470	17,649,147,000	134,346,496	13,434,649,600	76.12%	0.21%	0.14%	4.50%	13.44%	5.59%
**BAP8-104**	245,440,424	24,544,042,400	195,797,490	19,579,749,000	79.77%	0.19%	0.12%	4.83%	9.29%	5.80%
**BAP8-109**	174,914,674	17,491,467,400	138,556,555	13,855,655,500	79.21%	0.22%	0.13%	4.81%	9.71%	5.92%
**BAP8-110**	226,719,282	22,671,928,200	188,251,188	18,825,118,800	83.03%	0.13%	0.16%	4.72%	8.81%	3.14%
**BAP8-116**	177,767,074	17,776,707,400	142,848,226	14,284,822,600	80.36%	0.10%	0.14%	4.86%	8.79%	5.75%
**BAP8-121**	222,093,586	22,209,358,600	173,792,851	17,379,285,100	78.25%	0.10%	0.15%	4.66%	11.30%	5.54%
**BAP8-123**	193,190,134	19,319,013,400	160,096,068	16,009,606,800	82.87%	0.12%	0.14%	5.11%	6.18%	5.57%
**BAP8-126**	171,268,832	17,126,883,200	135,658,632	13,565,863,200	79.21%	0.14%	0.15%	4.94%	9.96%	5.60%
**BAP8-129**	211,822,726	21,182,272,600	175,703,867	17,570,386,700	82.95%	0.18%	0.15%	5.15%	5.96%	5.60%
**BAP8-130**	237,388,578	23,738,857,800	187,864,111	18,786,411,100	79.14%	0.15%	0.15%	4.83%	9.91%	5.82%
**BAP8-131**	200,623,154	20,062,315,400	156,944,242	15,694,424,200	78.23%	0.18%	0.15%	4.68%	11.23%	5.54%
**BAP8-136**	221,645,006	22,164,500,600	176,843,967	17,684,396,700	79.79%	0.20%	0.17%	4.88%	9.07%	5.90%
**BAP8-142**	170,217,914	17,021,791,400	131,390,510	13,139,051,000	77.19%	0.16%	0.16%	4.84%	12.21%	5.44%
**BAP8-165**	197,011,320	19,701,132,000	158,544,841	15,854,484,100	80.47%	0.16%	0.16%	5.02%	8.54%	5.64%
**BAP8-166**	159,287,506	15,928,750,600	124,729,402	12,472,940,200	78.30%	0.18%	0.16%	4.96%	10.78%	5.62%
**BAP8-172**	186,046,788	18,604,678,800	148,463,229	14,846,322,900	79.80%	0.11%	0.11%	5.12%	9.65%	5.20%
**BAP8-177**	205,479,680	20,547,968,000	165,671,443	16,567,144,300	80.63%	0.14%	0.12%	5.04%	8.74%	5.34%
**BAP8-178**	202,760,054	20,276,005,400	161,314,179	16,131,417,900	79.56%	0.15%	0.12%	4.98%	9.78%	5.41%
**BAP8-181**	173,014,260	17,301,426,000	139,808,535	13,980,853,500	80.81%	0.17%	0.12%	4.98%	8.53%	5.38%
**BAP8-183**	206,530,788	20,653,078,800	171,597,763	17,159,776,300	83.09%	0.12%	0.17%	5.45%	5.78%	5.39%
**BAP8-184**	206,805,894	20,680,589,400	167,802,957	16,780,295,700	81.14%	0.10%	0.18%	5.28%	8.07%	5.24%
**BAP8-186**	212,951,440	21,295,144,000	173,739,511	17,373,951,100	81.59%	0.10%	0.18%	5.27%	7.52%	5.34%
**BAP8-188**	184,596,062	18,459,606,200	148,364,082	14,836,408,200	80.37%	0.08%	0.17%	5.21%	8.80%	5.36%
**BAP8-195**	187,673,652	18,767,365,200	154,247,685	15,424,768,500	82.19%	0.07%	0.50%	4.81%	6.46%	5.98%
**BAP8-197**	223,009,638	22,300,963,800	178,888,939	17,888,893,900	80.22%	0.09%	0.49%	4.77%	8.39%	6.04%
**BAP8-198**	188,856,522	18,885,652,200	147,733,187	14,773,318,700	78.23%	0.09%	0.49%	4.74%	10.93%	5.52%
**BAP8-202**	160,915,716	16,091,571,600	125,803,400	12,580,340,000	78.18%	0.12%	0.51%	4.69%	10.83%	5.67%
**BAP8-203**	191,540,896	19,154,089,600	149,133,646	14,913,364,600	77.86%	0.06%	4.41%	4.56%	7.50%	5.60%
**BAP8-215**	171,722,564	17,172,256,400	134,772,982	13,477,298,200	78.48%	0.05%	4.40%	4.71%	6.78%	5.57%
**BAP8-234**	194,202,608	19,420,260,800	147,142,037	14,714,203,700	75.77%	0.05%	4.38%	4.81%	9.85%	5.14%
**BAP8-243**	184,942,354	18,494,235,400	144,254,912	14,425,491,200	78.00%	0.04%	4.39%	4.87%	7.39%	5.30%
**Total**	**12,568,199,794**	**1,256,819,979,400**	**10,019,751,062**	**1,001,975,106,200**	**79.71%**	**0.17%**	**0.52%**	**4.92%**	**9.07%**	**5.61%**
