# Supplementary figures and images for: SNP discovery and genetic mapping using genotyping by sequencing of whole genome genomic DNA from a pea RIL population
Source: BMC Genomics. 2016 Feb 18;17:121. doi: 10.1186/s12864-016-2447-2 (PMC4758021; doi:10.1186/s12864-016-2447-2)

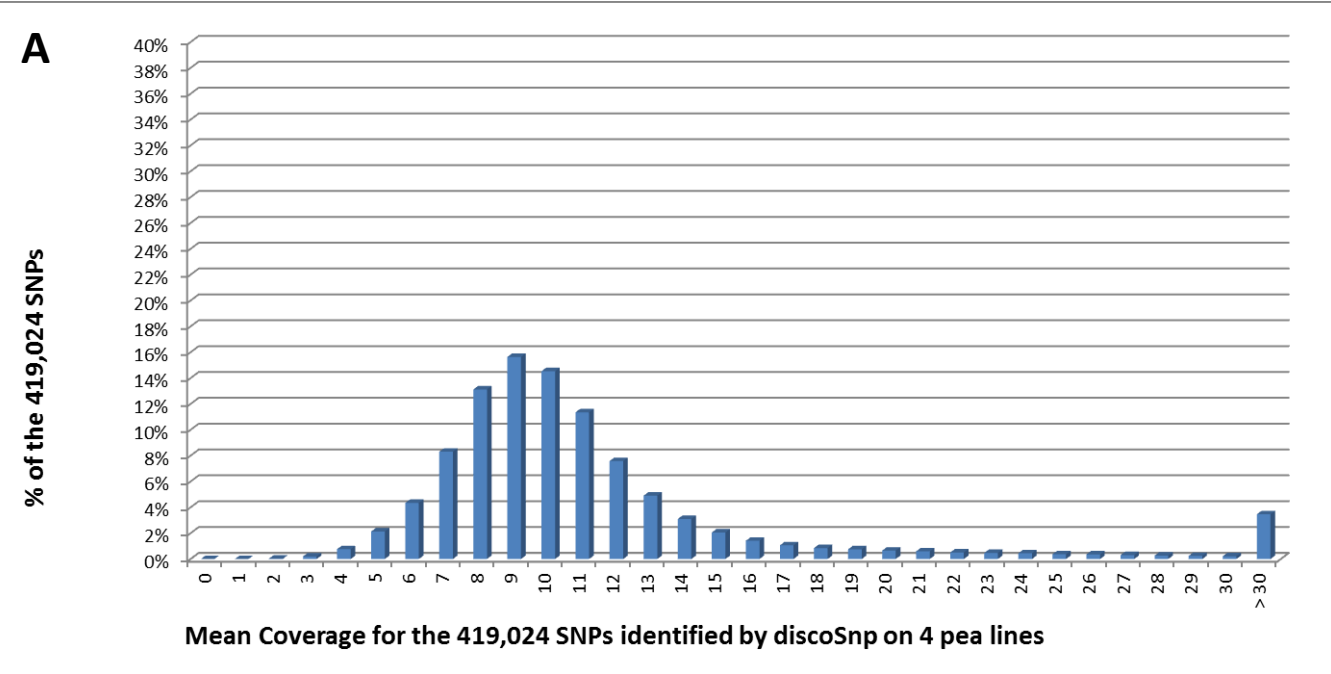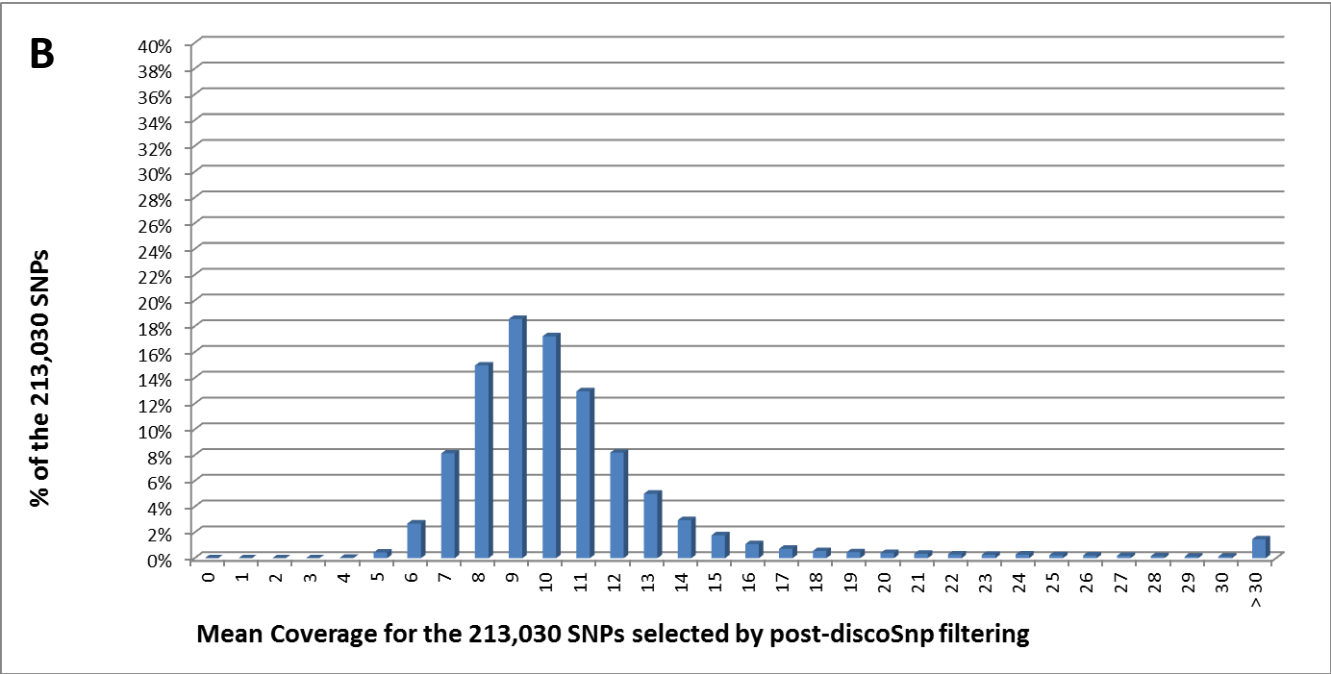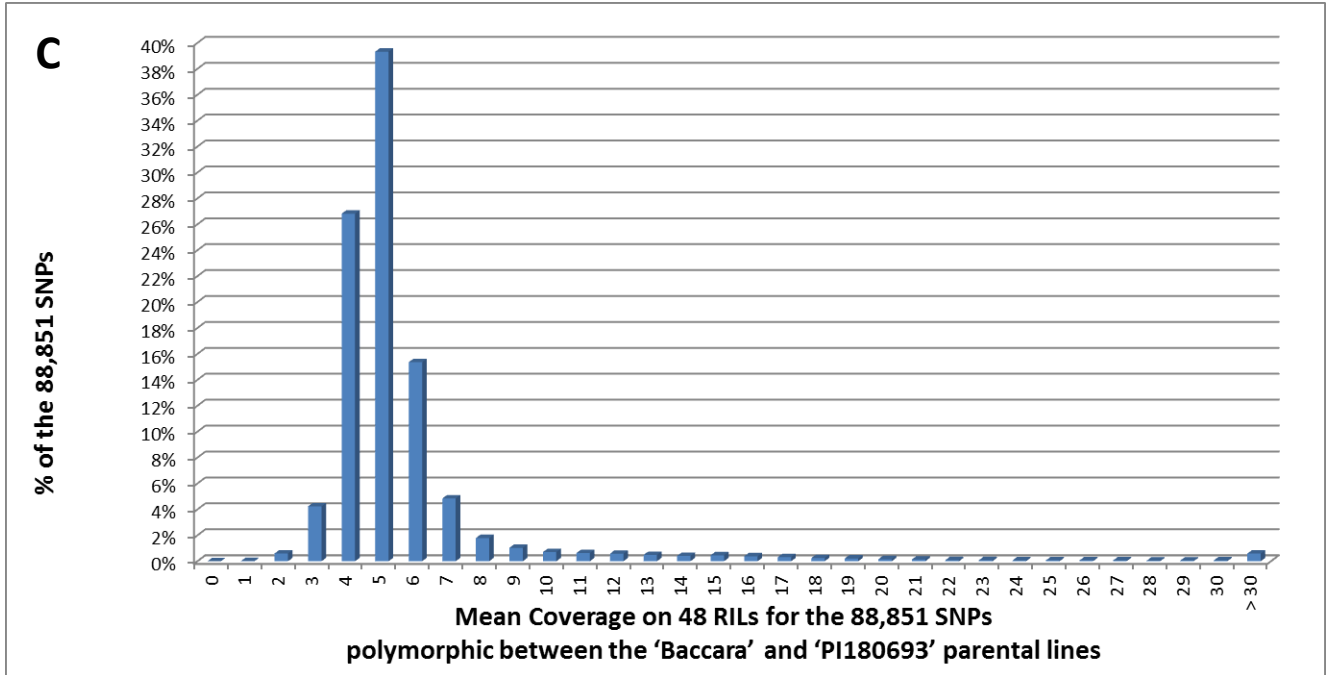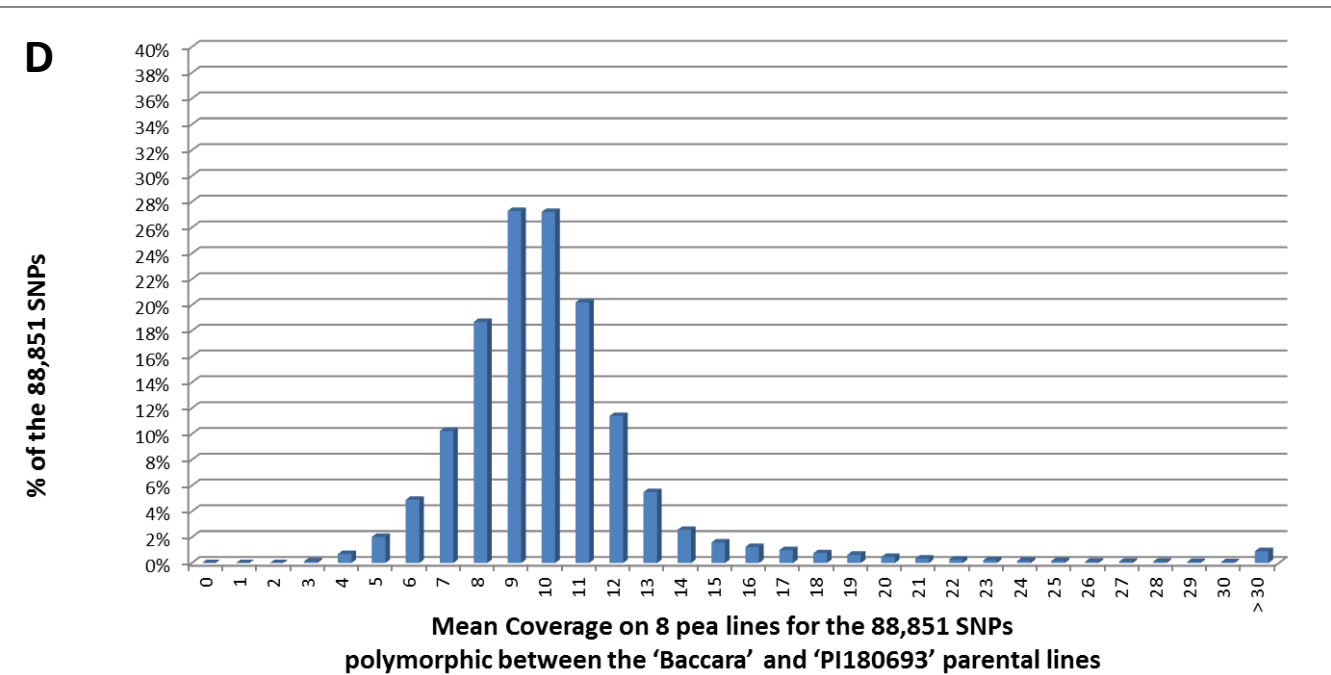

Supplement: Additional file 2: Figure S1. — Frequency histograms of SNP coverage. (A) corresponds to 419,024 SNPs identified by discoSnp on four pea lines, (B) to 213,030 SNPs selected after post discoSnp filtering on four pea lines, (C) to a selection of 88,851 polymorphic SNPs on 48 pea RILs, (D) to a selection of 88,851 polymorphic SNPs on eight pea lines. (PDF 267 kb) [file 12864_2016_2447_MOESM2_ESM.pdf]

LGI

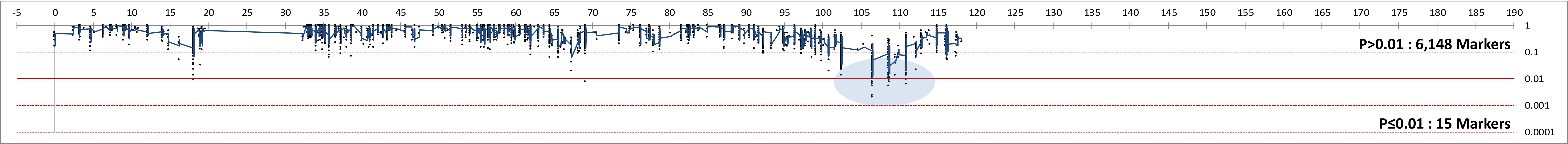

LGII

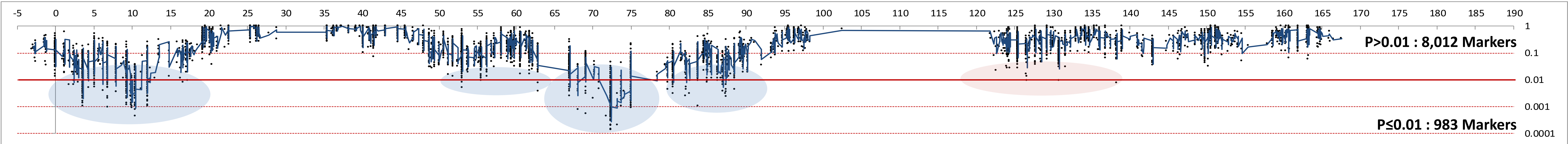

LGIII

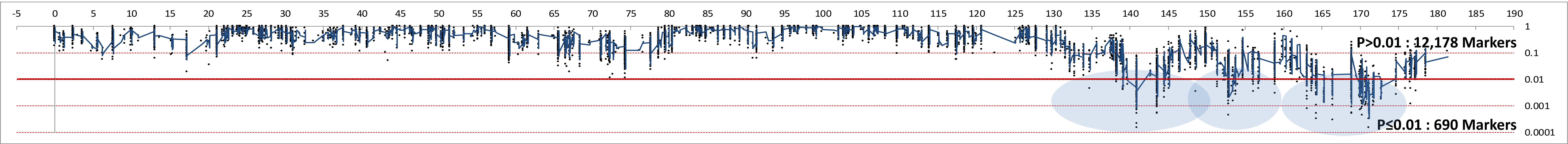

LGIV

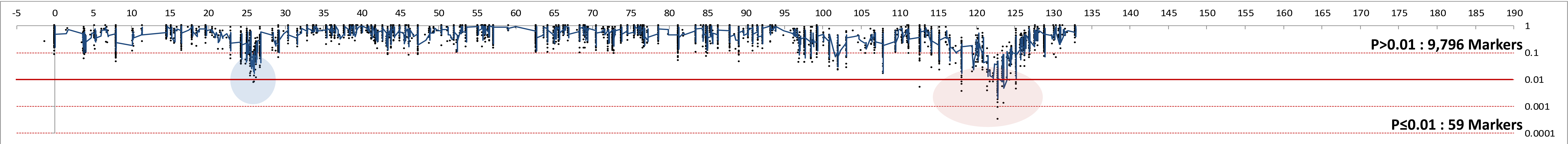

LGV

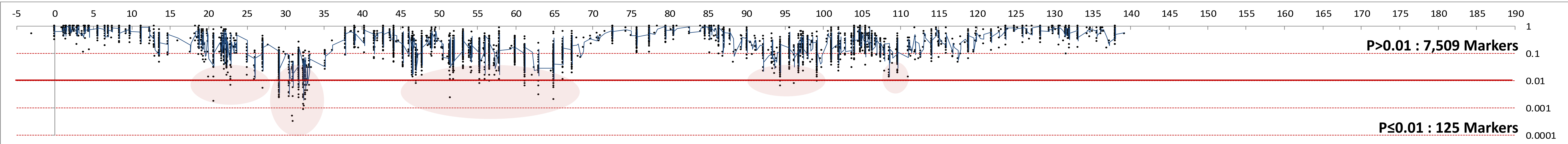

LGVI

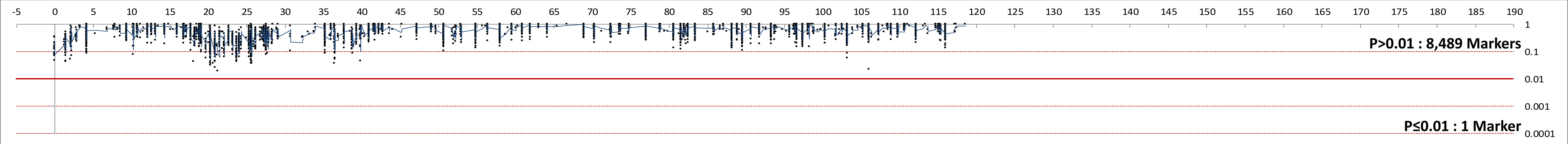

LGVII

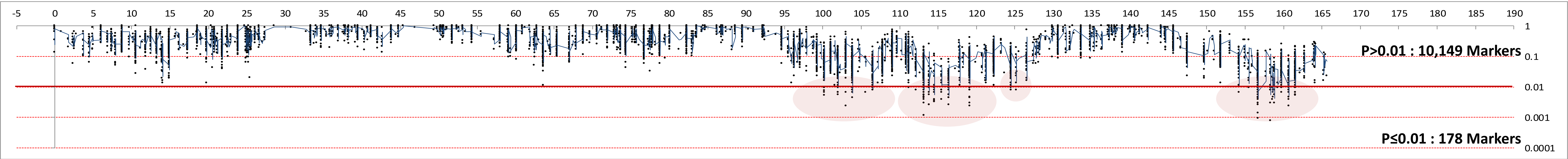

Supplement: Additional file 6: Figure S3. — Localisation and proportions of non-distorted (P > 0.01) and distorted (P < 0.01) markers along the linkage groups of the pea BP-WGGBS map. Red and blue ellipses indicate distorsions towards the ‘Baccara’ and ‘PI180693’ parental alleles, respectively. X axis is distance in cM, Y axis is Chi-square probability. (PDF 1114 kb) [file 12864_2016_2447_MOESM6_ESM.pdf]
