# Supplementary material for: SNP discovery and genetic mapping using genotyping by sequencing of whole genome genomic DNA from a pea RIL population
Source: BMC Genomics. 2016 Feb 18;17:121. doi: 10.1186/s12864-016-2447-2 (PMC4758021; doi:10.1186/s12864-016-2447-2)
Supplement: Additional file 3:Table S2. — Comparative genotyping of the 48 ‘Baccara’ x ‘PI180693’ RILs using 45 SNPs genotyped both through a GoldenGate® Assay (Duarte et al., 2014) and direct Whole Genome Genotyping by Sequencing (this study). SNP codes Ps0xxxxx and Ps0xxxxx_SNP_path_xxxxxx correspond to the GoldenGate® assay and to the direct sequencing assay, respectively. “A” corresponds to a ‘Baccara’ homozygous parental genotype, “B” to a ‘PI180693’ homozygous parental genotype, “H” to an heterozygous genotype, “-” indicates missing data. (PDF 332 kb) [file 12864_2016_2447_MOESM3_ESM.pdf]

	RIL Number																																														
SNP Code	Bap8_004	Bap8_006	Bap8_018	Bap8_024	Bap8_029	Bap8_037	Bap8_043	Bap8_047	Bap8_051	Bap8_056	Bap8_057	Bap8_060	Bap8_066	Bap8_070	Bap8_082	Bap8_090	Bap8_094	Bap8_099	Bap8_104	Bap8_109	Bap8_110	Bap8_116	Bap8_121	Bap8_123	Bap8_126	Bap8_129	Bap8_130	Bap8_131	Bap8_136	Bap8_142	Bap8_165	Bap8_166	Bap8_172	Bap8_177	Bap8_178	Bap8_181	Bap8_183	Bap8_184	Bap8_186	Bap8_188	Bap8_195	Bap8_197	Bap8_198	Bap8_202	Bap8_203	Bap8_215	Bap8_234	Bap8_243
Ps000027_Goldengate	B	A	B	A	A	B	A	A	B	A	B	A	B	B	A	A	B	A	A	A	A	B	B	B	B	B	B	A	B	A	A	A	B	A	-	A	B	B	A	A	A	A	B	B	A	A	A	B
Ps000027_WGGBS	H	A	B	A	A	B	A	A	B	A	B	A	B	B	H	A	B	A	A	A	A	H	B	B	B	B	B	H	B	A	A	A	B	A	B	A	B	B	-	A	A	A	B	B	A	A	A	B
Ps000078_Goldengate	B	B	A	A	B	A	B	A	A	A	A	B	B	A	A	A	B	A	A	B	A	B	A	B	A	B	B	B	A	A	A	B	B	B	A	A	B	B	B	A	A	-	A	A	A	B	B	B
Ps000078_WGGBS	B	B	A	A	B	A	B	A	A	A	A	B	H	A	H	A	B	A	A	B	H	B	A	B	A	B	B	B	A	A	H	B	B	B	A	A	B	B	B	A	A	B	-	A	A	B	B	B
Ps000204_Goldengate	-	B	A	B	A	B	A	B	B	-	B	A	B	A	A	A	A	B	B	B	B	A	A	B	B	A	A	B	A	B	B	A	B	B	B	A	B	B	A	B	B	B	A	B	B	B	B	A
Ps000204_WGGBS	A	H	A	B	A	B	A	B	B	B	B	A	-	A	A	A	A	B	B	B	B	A	A	B	B	A	H	B	A	B	B	A	H	B	B	A	B	B	A	B	B	B	A	B	B	B	B	A
Ps000291_Goldengate	-	B	A	B	B	A	B	A	A	B	A	B	A	A	B	B	A	A	A	B	A	A	A	B	B	A	-	B	B	A	-	A	H	-	-	B	B	A	B	B	B	A	H	A	A	A	B	B
Ps000291_WGGBS	A	H	A	B	B	A	H	A	A	B	A	B	A	A	B	B	A	A	A	B	A	A	A	B	B	A	A	B	B	A	A	-	H	A	A	B	B	A	B	B	B	A	H	A	A	A	B	B
Ps000333_Goldengate	B	B	B	B	A	A	B	A	A	A	B	A	B	A	B	B	A	-	A	A	A	A	B	A	A	A	A	A	B	B	A	B	B	B	B	A	B	A	A	A	B	B	H	A	-	B	A	-
Ps000333_WGGBS	B	B	B	B	H	A	B	A	A	H	B	A	B	A	B	B	A	B	A	A	A	A	B	H	A	A	A	A	B	B	A	B	B	B	B	A	B	A	A	A	B	B	H	-	A	B	A	A
Ps000349_Goldengate	FAILED on Goldengate																																														
Ps000349_WGGBS	B	A	A	A	A	B	B	A	B	B	B	B	A	B	A	A	B	B	A	A	B	A	B	B	H	B	B	B	B	B	B	B	H	B	B	B	B	B	B	B	B	B	B	B	B	B	H	B
Ps000364_Goldengate	B	B	A	B	A	A	A	A	A	A	B	A	B	A	B	B	A	-	A	A	A	B	B	A	A	A	A	A	B	B	A	B	B	A	A	A	B	A	A	A	B	B	A	B	A	B	A	B
Ps000364_WGGBS	B	B	A	B	H	A	A	A	A	A	B	A	B	A	B	B	A	B	A	A	A	B	B	A	A	A	A	H	B	B	A	B	B	A	A	A	B	A	A	A	B	B	A	B	A	B	A	H
Ps000478_Goldengate	A	A	A	A	A	A	B	B	A	A	A	A	B	A	B	A	A	B	A	B	A	A	A	A	A	B	A	A	A	A	A	A	A	B	A	A	B	B	B	A	B	A	B	B	B	B	A	A
Ps000478_WGGBS	A	A	A	A	A	A	B	B	A	A	A	A	B	H	B	A	A	B	A	B	H	A	A	-	A	B	A	A	A	A	A	A	H	B	A	A	-	B	H	A	B	A	B	B	B	B	A	A
Ps000480_Goldengate	A	B	B	B	A	A	B	B	A	A	B	A	A	A	A	A	B	B	B	A	A	B	A	B	A	A	A	B	A	A	A	A	A	A	A	A	B	B	B	B	A	B	B	A	B	B	A	B
Ps000480_WGGBS	A	B	B	B	A	H	B	-	A	A	B	A	A	A	A	A	B	B	B	A	A	B	A	B	A	A	A	B	A	A	A	-	B	A	A	A	B	B	B	B	A	B	B	A	B	B	A	H
Ps000491_Goldengate	B	A	B	B	B	B	A	A	B	B	A	A	A	-	A	A	A	B	A	A	A	A	A	A	A	A	B	A	B	A	A	B	B	B	A	A	B	A	A	A	A	B	B	A	A	A	A	B
Ps000491_WGGBS	B	A	B	B	B	B	A	A	B	B	A	A	A	B	A	A	H	B	A	A	A	A	A	A	A	A	B	A	B	A	A	B	-	B	A	H	B	A	A	A	A	B	B	A	A	A	A	B
Ps000505_Goldengate	A	B	A	B	A	B	B	B	B	B	B	B	A	A	A	A	A	B	B	A	B	A	A	B	B	B	A	A	A	A	A	B	A	B	B	B	B	B	A	B	A	B	B	A	A	A	A	B
Ps000505_WGGBS	A	B	A	B	A	B	B	B	B	B	B	B	A	A	-	A	A	B	B	A	B	A	A	B	B	B	A	A	A	A	H	B	H	B	B	B	B	B	A	-	A	-	B	A	A	A	A	B
Ps000528_Goldengate	A	B	A	A	A	B	B	B	B	A	B	B	A	A	A	A	A	B	B	A	B	A	B	B	B	B	A	A	A	A	A	A	A	B	H	A	B	B	B	A	A	B	B	A	B	A	A	B
Ps000528_WGGBS	H	B	H	A	A	B	B	B	B	A	B	B	A	A	A	A	A	B	B	A	B	A	B	B	B	B	A	A	A	A	A	-	A	B	H	H	B	B	B	-	A	B	B	A	B	A	A	B
Ps000604_Goldengate	A	B	A	B	B	B	A	B	B	A	B	B	B	A	B	B	A	A	B	B	B	A	A	B	A	B	B	B	A	B	B	A	B	B	B	B	B	B	-	B	B	B	A	A	B	B	B	B
Ps000604_WGGBS	A	B	A	B	B	B	A	B	H	A	B	B	B	A	B	B	A	A	B	B	B	A	A	B	A	B	B	B	A	B	B	A	B	B	B	B	B	B	B	-	B	B	A	A	B	B	B	B
Ps000614_Goldengate	A	B	A	A	B	A	A	A	A	A	A	B	A	A	A	A	B	A	A	B	B	-	B	A	A	B	A	A	B	A	A	A	B	A	A	A	B	A	A	A	A	B	B	A	B	A	B	B
Ps000614_WGGBS	A	B	H	A	B	A	A	A	A	A	A	B	A	A	A	A	B	A	A	B	B	B	B	A	A	H	A	A	B	A	A	A	B	A	A	A	B	A	A	A	A	B	H	A	B	A	B	B
Ps000629_Goldengate	A	B	B	A	A	A	B	B	A	A	B	B	A	A	A	A	B	B	A	B	A	A	A	B	B	A	A	B	A	A	A	B	A	B	B	A	B	A	B	B	B	B	A	B	B	A	B	B
Ps000629_WGGBS	H	B	B	A	A	A	B	B	A	A	B	B	A	A	A	A	B	B	A	B	H	A	A	B	B	A	A	B	A	A	A	B	A	-	B	A	B	A	H	B	-	B	A	B	H	A	B	B
Ps000638_Goldengate	B	A	B	A	A	A	B	A	B	A	A	B	B	A	A	A	B	-	A	B	A	A	B	B	A	B	B	A	-	A	A	A	A	A	A	A	B	B	B	A	B	B	B	B	B	A	A	A
Ps000638_WGGBS	B	A	B	A	A	A	B	A	B	A	A	B	B	A	H	A	B	A	A	B	A	H	B	B	A	B	B	A	A	A	A	A	-	-	A	A	B	B	B	A	B	B	B	B	B	A	A	A
Ps000642_Goldengate	B	A	B	B	A	B	A	A	B	A	B	A	B	B	A	A	B	B	A	A	A	B	A	B	B	A	B	A	B	A	A	B	B	B	B	A	B	B	A	A	A	-	B	B	A	A	A	B
Ps000642_WGGBS	B	A	B	B	A	B	A	A	B	H	B	A	B	B	-	A	-	B	H	H	A	B	A	B	B	A	B	A	B	A	A	B	H	B	B	-	B	B	A	A	A	B	B	B	A	A	A	B
Ps000656_Goldengate	B	B	B	-	A	A	B	A	B	A	A	B	B	A	A	A	B	-	A	A	A	A	B	B	A	B	B	A	-	A	A	A	B	A	A	A	B	B	B	A	B	B	B	B	B	A	B	B
Ps000656_WGGBS	B	B	B	H	A	A	B	A	B	A	A	B	B	A	A	A	B	A	A	A	A	A	B	B	A	B	B	A	B	A	A	A	B	A	A	A	B	B	B	A	B	B	B	B	B	A	B	B
Ps000658_Goldengate	A	B	B	A	A	A	B	B	A	A	B	B	A	A	A	A	B	B	A	A	A	A	A	B	B	A	A	B	A	A	A	B	A	A	A	A	B	A	B	B	B	B	A	B	B	A	A	B
Ps000658_WGGBS	A	B	B	A	-	A	B	B	A	A	B	B	A	-	A	A	B	B	A	-	A	A	A	B	H	A	A	B	A	H	A	B	A	A	A	-	B	A	B	B	B	B	A	B	B	A	A	B
Ps000690_Goldengate	A	B	B	B	A	A	B	B	B	A	B	B	A	A	B	B	B	B	B	A	B	B	A	B	A	A	A	A	A	A	A	A	A	A	B	A	H	B	B	B	A	B	B	A	B	A	A	B
Ps000690_WGGBS	A	B	B	B	A	A	B	-	B	A	B	-	A	A	B	B	B	B	B	A	B	B	A	B	A	A	A	A	A	A	A	A	A	A	B	-	H	B	B	B	A	B	-	H	H	H	A	B
Ps000734_Goldengate	H	B	A	B	A	B	A	B	B	-	B	A	B	A	A	A	A	B	B	B	B	A	A	B	B	A	A	B	A	B	-	A	B	B	B	A	B	B	A	B	B	B	A	B	B	B	B	A
Ps000734_WGGBS	H	B	A	B	A	B	A	B	B	A	-	A	B	A	A	H	A	-	B	B	B	A	H	B	B	A	A	B	A	B	B	A	B	B	-	A	B	B	A	B	B	B	A	B	B	B	B	A
Ps000741_Goldengate	A	A	B	A	A	A	B	A	B	A	A	A	B	A	A	A	A	A	A	B	A	A	A	B	A	B	B	A	-	A	A	A	A	B	A	A	B	B	B	A	B	A	B	B	B	A	A	A
Ps000741_WGGBS	A	A	B	A	A	A	B	A	B	A	A	A	B	A	A	A	A	A	A	B	A	A	A	B	A	B	B	-	A	A	A	A	A	B	A	A	B	B	B	A	B	A	B	B	B	A	A	A
Ps000768_Goldengate	FAILED on Goldengate																																														
Ps000768_WGGBS	A	A	A	A	A	B	B	B	A	A	B	B	A	A	A	A	B	B	H	A	A	A	A	B	B	A	A	B	A	B	B	-	A	A	B	B	H	A	B	B	B	B	H	B	B	B	B	A
Ps000770_Goldengate	A	B	A	A	A	A	B	A	B	B	B	B	A	A	A	A	A	A	B	A	A	B	B	B	B	A	B	B	A	A	-	A	B	A	A	A	A	B	A	B	B	B	H	B	B	B	A	A
Ps000770_WGGBS	A	B	A	A	A	A	B	H	B	B	B	B	A	A	A	A	A	A	B	A	A	B	B	B	B	A	B	B	A	A	B	A	B	A	A	A	A	B	A	B	B	B	H	B	B	B	A	A
Ps000813_Goldengate	A	A	A	B	B	A	A	A	B	B	A	A	A	A	A	B	B	B	A	A	B	A	B	B	A	B	B	B	A	A	B	A	A	A	A	A	A	A	A	-	B	B	B	B	B	A	A	B
Ps000813_WGGBS	A	A	A	B	B	A	A	A	B	B	A	A	A	H	A	B	B	B	A	H	B	A	B	B	A	B	B	B	A	A	B	A	A	A	A	A	H	H	A	A	B	B	B	B	B	A	A	B
Ps000819_Goldengate	FAILED on Goldengate																																														
Ps000819_WGGBS	A	A	A	B	B	H	A	A	B	B	A	A	A	B	A	B	B	B	A	A	A	A	B	A	A	B	B	B	A	A	B	A	-	A	A	-	A	A	H	A	B	B	B	B	B	A	A	B
Ps000836_Goldengate	A	A	B	B	A	A	B	B	A	A	A	A	A	B	B	A	A	B	A	B	A	A	A	A	A	B	A	A	A	A	A	A	A	B	A	A	B	B	B	A	B	A	B	B	B	A	A	A
Ps000836_WGGBS	H	A	B	B	A	A	B	B	A	A	A	A	H	-	H	A	A	B	A	B	A	A	A	-	A	B	A	A	A	A	A	A	A	B	A	A	B	B	B	A	B	A	H	B	B	A	A	A
Ps000922_Goldengate	A	B	A	B	A	A	A	A	B	A	A	H	A	A	B	B	A	A	B	A	A	B	B	B	A	B	B	B	A	A	B	A	A	A	A	A	A	B	A	B	B	B	B	A	B	A	A	B
Ps000922_WGGBS	A	B	-	B	A	A	A	A	B	A	A	H	A	A	H	B	A	A	B	H	A	B	B	B	A	B	B	B	A	A	B	-	-	A	A	A	A	B	A	B	B	B	B	A	H	A	A	B
Ps000972_Goldengate	A	A	A	A	A	A	B	H	A	A	A	A	B	A	B	A	A	B	A	B	A	A	A	A	A	B	A	A	A	A	A	A	A	B	A	A	B	B	B	A	B	A	B	B	B	B	A	A
Ps000972_WGGBS	A	A	A	A	A	A	B	H	A	A	A	H	H	A	B	A	A	B	A	B	A	A	A	A	A	B	A	A	A	A	A	A	A	B	H	A	B	B	B	A	B	A	B	B	B	B	A	A
Ps001120_Goldengate	A	A	A	B	B	A	A	B	B	B	A	A	A	B	A	B	B	B	A	A	A	B	B	A	A	B	B	B	A	A	B	A	A	A	A	A	A	A	A	-	B	B	B	B	B	A	A	B
Ps001120_WGGBS	A	A	A	-	B	A	A	B	B	B	A	A	A	B	A	B	B	B	A	A	A	B	B	A	A	B	B	B	A	H	H	A	A	A	A	A	A	A	A	A	B	H	B	B	B	A	A	B
Ps001128_Goldengate	FAILED on Goldengate																																														
Ps001128_WGGBS	A	B	A	H	A	A	B	A	B	B	B	B	A	A	H	B	A	A	A	A	A	B	H	B	B	A	B	B	A	A	B	A	B	A	A	A	A	B	A	B	B	B	B	A	B	B	A	A
Ps001405_Goldengate	B	A	B	A	A	B	A	A	B	A	B	A	B	B	A	A	B	A	A	A	A	B	B	B	B	B	A	A	B	A	A	A	A	A	H	A	B	B	A	A	A	A	B	B	A	A	A	A
Ps001405_WGGBS	B	A	B	A	A	B	A	A	B	A	B	A	B	B	A	A	B	A	A	A	A	-	B	H	-	B	A	A	-	A	A	A	A	-	H	A	B	B	A	A	A	A	B	B	A	A	A	A
Ps001424_Goldengate	B	B	B	B	A	A	B	A	A	A	A	A	A	A	B	B	A	B	A	B	H	A	B	A	A	A	B	B	B	B	A	A	B	B	B	A	A	B	B	A	A	A	B	A	B	A	B	A
Ps001424_WGGBS	B	B	B	B	A	A	B	-	A	A	A	A	A	A	H	B	A	B	A	-	H	A	B	A	A	A	B	B	-	B	A	H	B	B	-	A	A	B	B	A	A	A	B	A	B	A	B	A
Ps001536_Goldengate	B	A	B	A	A	B	A	A	A	A	B	A	B	A	A	A	B	A	A	A	A	B	B	B	B	B	A	B	B	A	A	A	A	B	H	A	B	B	A	A	A	A	A	B	A	A	A	A
Ps001536_WGGBS	B	A	-	A	A	B	A	A	A	A	B	A	B	A	A	H	B	A	A	H	A	-	B	B	B	B	A	B	B	A	A	A	A	B	H	A	B	B	A	A	A	A	A	B	A	A	A	A
Ps001562_Goldengate	B	B	A	A	A	A	A	A	A	B	B	A	B	A	A	A	A	A	A	A	B	B	B	B	A	B	B	B	B	B	-	A	A	A	A	A	B	-	A	A	A	A	A	B	-	A	A	A
Ps001562_WGGBS	B	B	A	A	A	A	A	-	A	B	B	A	B	A	-	A	A	A	A	A	B	B	H	B	A	B	B	B	B	B	B	A	A	A	H	A	B	A	A	A	A	A	A	-	B	A	A	H
Ps001569_Goldengate	A	A	B	A	A	A	B	B	A	A	A	A	B	B	B	A	A	B	A	B	A	A	A	A	A	B	A	A	A	A	B	A	A	B	A	A	B	B	B	A	B	A	B	B	B	B	A	A
Ps001569_WGGBS	A	A	B	A	A	A	B	B	A	A	A	A	B	B	B	A	A	-	A	B	A	A	A	A	A	B	A	A	A	A	B	A	H	B	A	A	B	B	B	A	B	A	B	B	H	B	A	A
Ps001582_Goldengate	B	B	A	A	A	A	B	A	B	B	B	A	B	A	B	B	A	A	A	A	B	B	A	B	A	B	A	A	A	B	B	A	A	A	A	A	B	B	A	A	A	A	A	A	A	A	A	A
Ps001582_WGGBS	B	B	A	H	A	A	H	H	B	B	B	A	B	A	B	B	A	A	A	A	B	B	A	B	A	B	A	A	A	B	B	H	A	A	A	A	B	B	A	A	A	A	A	A	A	A	A	A
Ps001641_Goldengate	FAILED on Goldengate																																														
Ps001641_WGGBS	B	B	A	A	A	A	B	A	B	B	B	H	B	A	B	B	A	A	H	A	A	B	A	B	B	B	A	B	A	B	B	A	A	H	A	-	H	A	A	A	A	A	A	A	A	B	A	B
Ps001645_Goldengate	B	B	A	A	B	A	B	A	A	A	A	B	B	A	A	A	B	A	A	B	A	B	B	B	A	B	B	B	B	B	A	B	B	B	A	A	B	B	B	A	B	H	A	A	A	B	A	B
Ps001645_WGGBS	B	B	A	A	B	A	B	A	A	-	H	B	B	A	A	-	B	A	A	B	A	B	B	-	A	B	B	B	B	B	A	B	B	B	A	A	B	B	B	A	B	H	-	A	A	B	A	B
Ps001692_Goldengate	B	A	B	A	A	B	A	A	B	A	B	A	B	B	A	A	B	A	A	A	A	B	B	B	B	B	A	A	B	A	A	A	A	A	-	A	B	B	A	A	A	A	B	B	A	A	A	A
Ps001692_WGGBS	B	A	B	A	A	B	A	A	B	A	B	A	B	B	A	-	B	A	A	A	A	B	B	B	B	B	A	A	B	A	A	A	A	H	A	A	B	B	A	A	A	A	B	B	A	A	A	H
Ps001698_Goldengate	B	-	B	B	B	B	B	B	B	B	B	A	A	B	B	B	B	A	B	B	B	B	B	B	A	B	A	B	A	B	B	A	A	B	B	A	B	B	B	B	A	B	H	A	B	A	A	A
Ps001698_WGGBS	B	B	B	B	B	B	B	B	B	B	B	A	A	B	B	B	B	-	B	B	B	B	B	B	A	B	A	B	A	H	B	A	A	B	B	-	H	B	B	B	A	B	H	A	B	A	A	A
Ps001744_Goldengate	B	B	B	B	B	A	A	A	B	B	A	B	B	-	A	A	B	A	B	B	B	B	A	A	B	A	B	B	B	A	A	A	B	A	A	B	A	B	B	B	B	A	B	A	A	B	A	A
Ps001744_WGGBS	B	B	B	B	-	A	A	A	B	B	A	B	B	B	A	A	B	A	B	B	B	B	A	A	B	A	B	B	B	A	A	A	B	A	A	B	A	B	B	B	B	A	B	A	A	B	A	A
Ps001762_Goldengate	FAILED on Goldengate																																														
Ps001762_WGGBS	B	A	B	B	B	A	B	B	B	B	A	B	A	A	A	B	H	A	A	H	A	B	A	A	A	A	B	B	A	A	A	A	A	A	A	-	A	H	B	A	-	B	A	B	B	B	B	B
Ps001827_Goldengate	B	A	B	A	A	B	B	B	B	B	A	B	B	-	A	A	B	A	B	B	A	A	B	A	B	A	B	B	A	-	A	A	B	A	A	A	-	B	A	B	B	A	B	B	A	B	A	B
Ps001827_WGGBS	B	A	B	A	A	B	B	B	B	-	A	B	B	-	A	A	B	-	B	B	H	A	B	H	B	A	H	B	A	A	-	A	B	A	A	A	B	H	A	B	B	A	B	B	A	B	A	B
Ps001903_Goldengate	B	B	B	B	B	A	A	A	B	A	A	B	B	B	A	A	B	A	B	A	A	B	B	A	B	A	B	A	A	A	A	A	B	A	A	A	A	B	A	A	B	A	-	A	A	A	B	A
Ps001903_WGGBS	B	B	B	B	B	A	A	A	B	A	A	H	B	B	A	A	B	A	B	A	A	B	B	A	B	-	B	-	-	H	A	A	B	A	A	A	A	B	A	A	B	A	A	A	A	A	B	A
