# Supplementary material for: SNP discovery and genetic mapping using genotyping by sequencing of whole genome genomic DNA from a pea RIL population
Source: BMC Genomics. 2016 Feb 18;17:121. doi: 10.1186/s12864-016-2447-2 (PMC4758021; doi:10.1186/s12864-016-2447-2)
Supplement: Additional file 7: Figure S2. — Colinearity of 914 markers common to the BP-WGGBS (left) and BP-Duarte (right) maps with the Duarte et al. (2014) reference consensus map (middle). (PDF 337 kb) [file 12864_2016_2447_MOESM7_ESM.pdf]

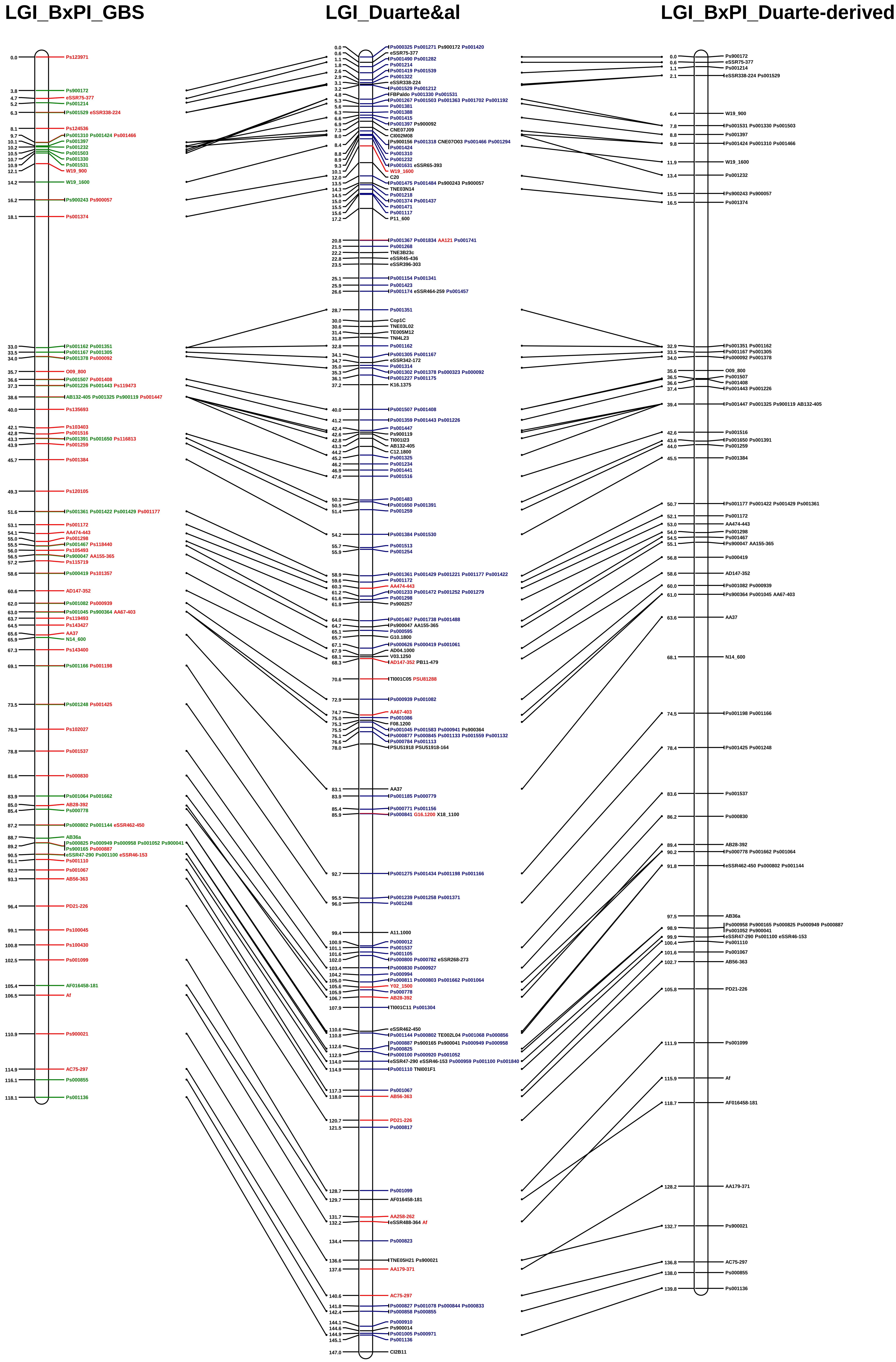
LGI_BxPI_GBS
LGI_Duarte&al
LGI_BxPI_Duarte-derived
Ps123971
Ps900172
eSSR75-377
Ps001214
Ps001529 eSSR338-224
Ps124536
Ps001310 Ps001424 Ps001466
Ps001397
Ps001232
Ps001503
Ps001330
Ps001531
W19_900
W19_1600
Ps900243 Ps900057
Ps001374
Ps001162 Ps001351
Ps001167 Ps001305
Ps001378 Ps000092
O09_800
Ps001507 Ps001408
Ps001226 Ps001443 Ps119473
AB132-405 Ps001325 Ps900119 Ps001447
Ps135693
Ps103403
Ps001516
Ps001391 Ps001650 Ps116813
Ps001259
Ps001384
Ps120105
Ps001361 Ps001422 Ps001429 Ps001177
Ps001172
AA474-443
Ps001298
Ps001467 Ps118440
Ps105493
Ps900047 AA155-365
Ps115719
Ps000419 Ps101357
AD147-352
Ps001082 Ps000939
Ps001045 Ps900364 AA67-403
Ps119493
Ps143427
AA37
N14_600
Ps143400
Ps001166 Ps001198
Ps001248 Ps001425
Ps102027
Ps001537
Ps000830
Ps001064 Ps001662
AB28-392
Ps000778
Ps000802 Ps001144 eSSR462-450
AB36a
Ps000825 Ps000949 Ps000958 Ps001052 Ps900041
Ps900165 Ps000887
eSSR47-290 Ps001100 eSSR46-153
Ps001110
Ps001067
AB56-363
PD21-226
Ps100045
Ps100430
Ps001099
AF016458-181
Af
Ps900021
AC75-297
Ps000855
Ps001136
Ps000325 Ps001271 Ps900172 Ps001420
eSSR75-377
Ps001490 Ps001282
Ps001214
Ps001419 Ps001539
Ps001322
eSSR338-224
Ps001529 Ps001212
FBPaldo Ps001330 Ps001531
Ps001267 Ps001503 Ps001363 Ps001702 Ps001192
Ps001381
Ps001388
Ps001415
Ps001397 Ps900092
CNE07J09
CI002M08
Ps900156 Ps001318 CNE07O03 Ps001466 Ps001294
Ps001424
Ps001310
Ps001232
Ps001631 eSSR65-393
W19_1600
C20
Ps001475 Ps001484 Ps900243 Ps900057
TNE03N14
Ps001218
Ps001374 Ps001437
Ps001471
Ps001117
P11_600
Ps001367 Ps001834 AA121 Ps001741
Ps001268
TNE3B23c
eSSR45-436
eSSR396-303
Ps001154 Ps001341
Ps001423
Ps001174 eSSR464-259 Ps001457
Ps001351
Cop1C
TNE03L02
TE005M12
TNI4L23
Ps001162
Ps001305 Ps001167
eSSR342-172
Ps001314
Ps001302 Ps001378 Ps000323 Ps000092
Ps001227 Ps001175
K16.1375
Ps001507 Ps001408
Ps001359 Ps001443 Ps001226
Ps001447
Ps900119
TI001I23
AB132-405
C12.1800
Ps001325
Ps001234
Ps001441
Ps001516
Ps001483
Ps001650 Ps001391
Ps001259
Ps001384 Ps001530
Ps001513
Ps001254
Ps001361 Ps001429 Ps001221 Ps001177 Ps001422
Ps001172
AA474-443
Ps001233 Ps001472 Ps001252 Ps001279
Ps001298
Ps900257
Ps001467 Ps001738 Ps001488
Ps900047 AA155-365
Ps000595
G10.1800
Ps000626 Ps000419 Ps001061
AD04.1000
V03.1250
AD147-352 PB11-479
TI001C05 PSU81288
Ps000939 Ps001082
AA67-403
Ps001086
F08.1200
Ps001045 Ps001583 Ps000941 Ps900364
Ps000877 Ps000845 Ps001133 Ps001559 Ps001132
Ps000784 Ps001113
PSU51918 PSU51918-164
AA37
Ps001185 Ps000779
Ps000771 Ps001156
Ps000841 G16.1200 X18_1100
Ps001275 Ps001434 Ps001198 Ps001166
Ps001239 Ps001258 Ps001371
Ps001248
A11.1000
Ps000012
Ps001537
Ps001105
Ps000800 Ps000782 eSSR268-273
Ps000830 Ps000927
Ps000994
Ps000811 Ps000803 Ps001662 Ps001064
Y02_1500
Ps000778
AB28-392
TI001C11 Ps001304
eSSR462-450
Ps001144 Ps000802 TE002L04 Ps001068 Ps000856
Ps000887 Ps900165 Ps900041 Ps000949 Ps000958
Ps000825
Ps000100 Ps000920 Ps001052
eSSR47-290 eSSR46-153 Ps000959 Ps001100 Ps001840
Ps001110 TNI001F1
Ps001067
AB56-363
PD21-226
Ps000817
Ps001099
AF016458-181
AA258-262
eSSR488-364 Af
Ps000823
TNE05H21 Ps900021
AA179-371
AC75-297
Ps000827 Ps001078 Ps000844 Ps000833
Ps000858 Ps000855
Ps000910
Ps900014
Ps001005 Ps000971
Ps001136
CI2B11
Ps900172
eSSR75-377
Ps001214
eSSR338-224 Ps001529
W19_900
Ps001531 Ps001330 Ps001503
Ps001397
Ps001424 Ps001310 Ps001466
W19_1600
Ps001232
Ps900243 Ps900057
Ps001374
Ps001351 Ps001162
Ps001167 Ps001305
Ps000092 Ps001378
O09_800
Ps001507
Ps001408
Ps001443 Ps001226
Ps001447 Ps001325 Ps900119 AB132-405
Ps001516
Ps001650 Ps001391
Ps001259
Ps001384
Ps001177 Ps001422 Ps001429 Ps001361
Ps001172
AA474-443
Ps001298
Ps001467
Ps900047 AA155-365
Ps000419
AD147-352
Ps001082 Ps000939
Ps900364 Ps001045 AA67-403
AA37
N14_600
Ps001198 Ps001166
Ps001425 Ps001248
Ps001537
Ps000830
AB28-392
Ps000778 Ps001662 Ps001064
eSSR462-450 Ps000802 Ps001144
AB36a
Ps000958 Ps900165 Ps000825 Ps000949 Ps000887
Ps001052 Ps900041
eSSR47-290 Ps001100 eSSR46-153
Ps001110
Ps001067
AB56-363
PD21-226
Ps001099
Af
AF016458-181
AA179-371
Ps900021
AC75-297
Ps000855
Ps001136

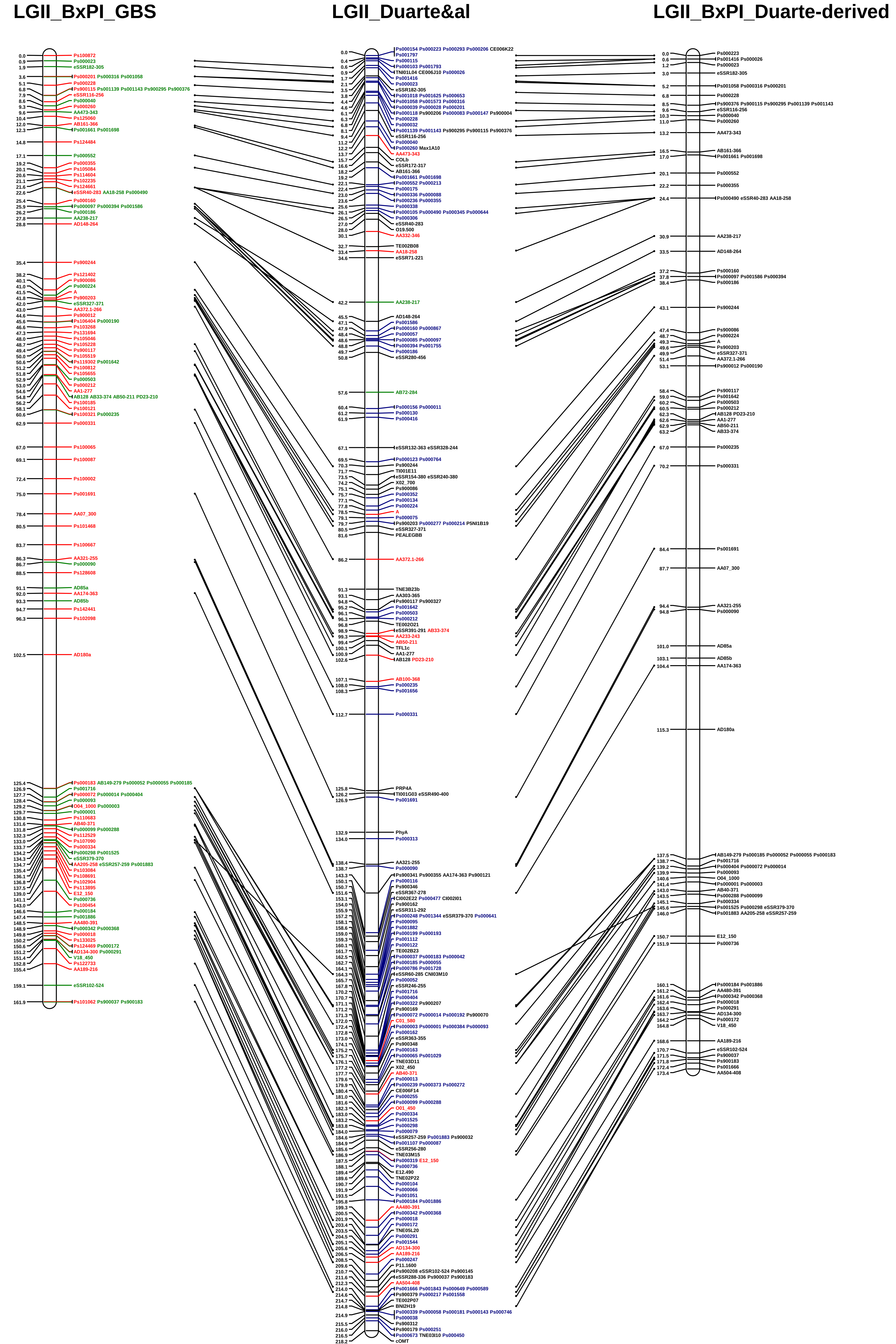
LGII_BxPI_GBS
LGII_Duarte&al
LGII_BxPI_Duarte-derived

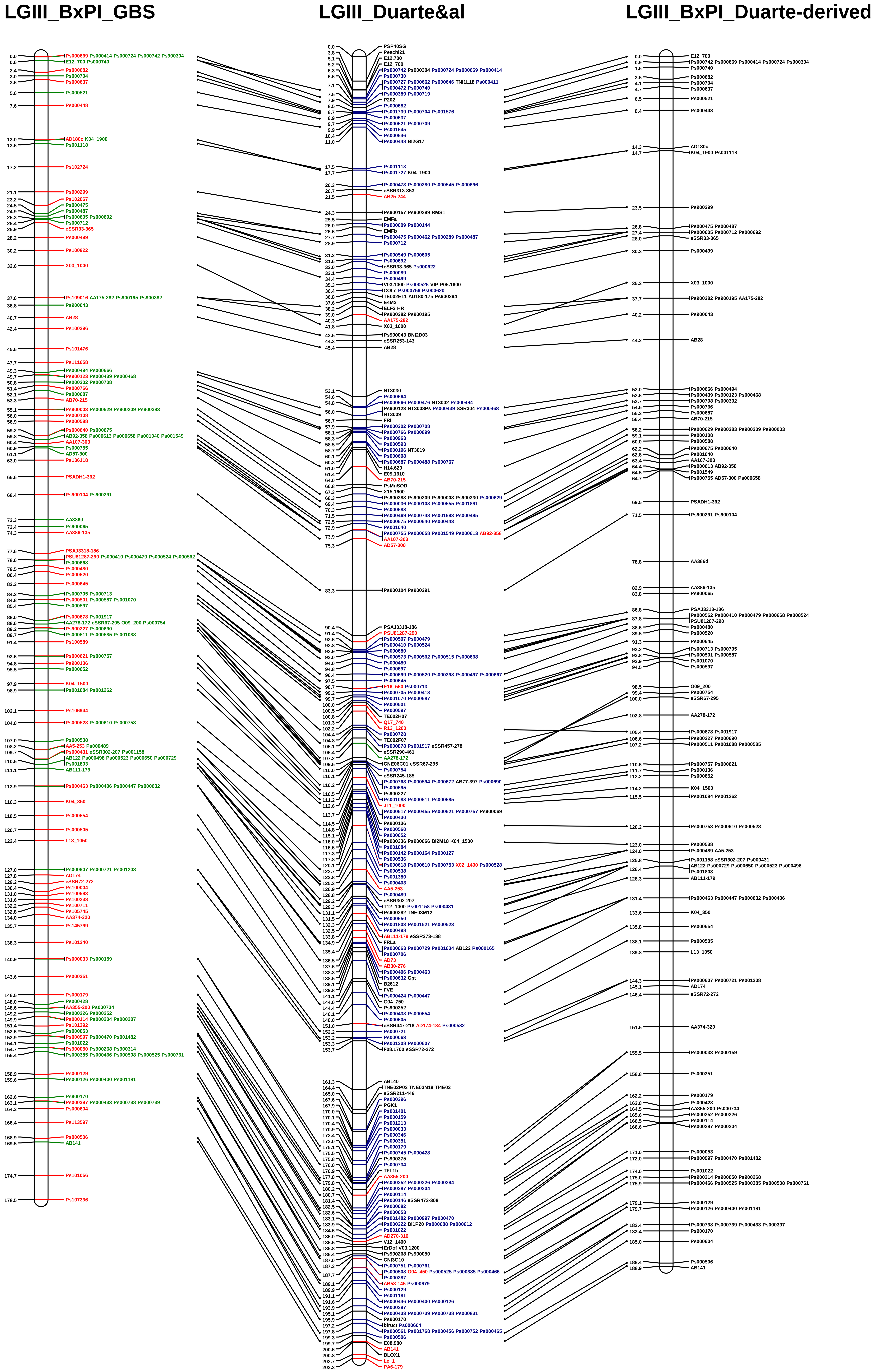
LGIII_BxPI_GBS
LGIII_Duarte&al
LGIII_BxPI_Duarte-derived

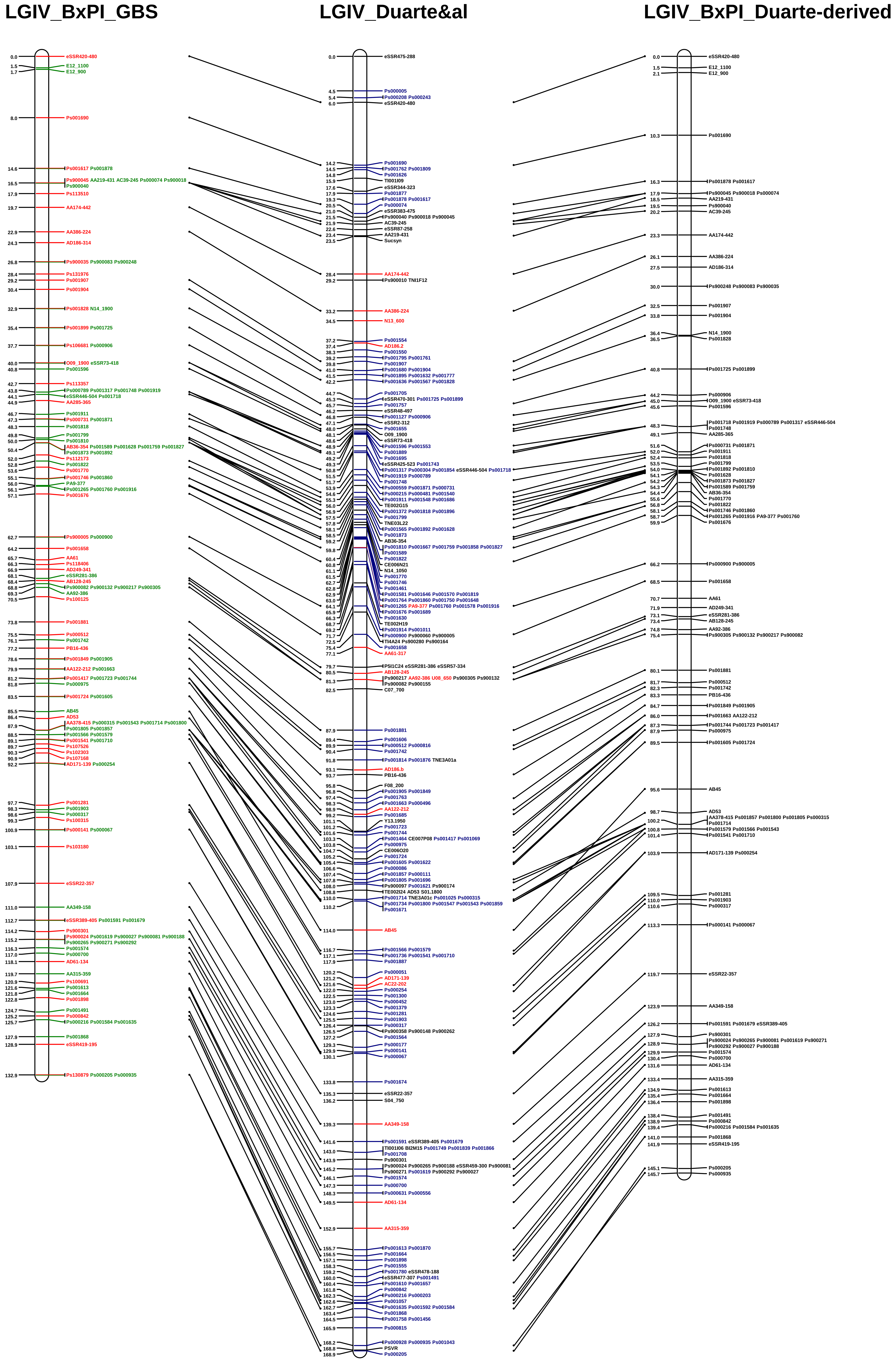

LGIV_BxPI_GBS
0.0 eSSR420-480
1.5 E12_1100
1.7 E12_900
8.0 Ps001690
14.6 Ps001617 Ps001878
16.5 Ps900045 AA219-431 AC39-245 Ps000074 Ps900018
Ps900040
17.9 Ps113510
19.7 AA174-442
22.9 AA386-224
24.3 AD186-314
26.8 Ps900035 Ps900083 Ps900248
28.4 Ps131976
29.2 Ps001907
30.4 Ps001904
32.9 Ps001828 N14_1900
35.4 Ps001899 Ps001725
37.7 Ps106681 Ps000906
40.0 O09_1900 eSSR73-418
40.8 Ps001596
42.7 Ps113357
43.8 Ps000789 Ps001317 Ps001748 Ps001919
44.1 eSSR446-504 Ps001718
44.9 AA285-365
46.7 Ps001911
47.3 Ps000731 Ps001871
48.3 Ps001818
49.8 Ps001799
50.0 Ps001810
50.4 AB36-354 Ps001589 Ps001628 Ps001759 Ps001827
Ps001873 Ps001892
52.0 Ps112173
52.8 Ps001822
53.6 Ps001770
55.1 Ps001746 Ps001860
56.0 PA9-377
56.1 Ps001265 Ps001760 Ps001916
57.1 Ps001676
62.7 Ps900005 Ps000900
64.2 Ps001658
65.7 AA61
66.3 Ps118406
66.9 AD249-341
68.1 eSSR281-386
68.4 AB128-245
68.8 Ps900082 Ps900132 Ps900217 Ps900305
69.3 AA92-386
70.5 Ps100125
73.8 Ps001881
75.5 Ps000512
76.1 Ps001742
77.2 PB16-436
78.6 Ps001849 Ps001905
79.9 AA122-212 Ps001663
81.2 Ps001417 Ps001723 Ps001744
81.8 Ps000975
83.5 Ps001724 Ps001605
85.5 AB45
86.4 AD53
87.9 AA378-415 Ps000315 Ps001543 Ps001714 Ps001800
Ps001805 Ps001857
88.5 Ps001566 Ps001579
89.1 Ps001541 Ps001710
89.7 Ps107526
90.3 Ps102303
90.9 Ps107168
92.2 AD171-139 Ps000254
97.7 Ps001281
98.3 Ps001903
98.6 Ps000317
99.3 Ps100315
100.9 Ps000141 Ps000067
103.1 Ps103180
107.9 eSSR22-357
111.0 AA349-158
112.7 eSSR389-405 Ps001591 Ps001679
114.2 Ps900301
115.2 Ps900024 Ps001619 Ps900027 Ps900081 Ps900188
Ps900265 Ps900271 Ps900292
116.3 Ps001574
117.0 Ps000700
118.1 AD61-134
119.7 AA315-359
120.9 Ps100691
121.6 Ps001613
121.8 Ps001664
122.8 Ps001898
124.7 Ps001491
125.2 Ps000842
125.7 Ps000216 Ps001584 Ps001635
127.9 Ps001868
128.9 eSSR419-195
132.9 Ps130879 Ps000205 Ps000935
LGIV_Duarte&al
0.0 eSSR475-288
4.5 Ps000005
5.4 Ps000208 Ps000243
6.0 eSSR420-480
14.2 Ps001690
14.5 Ps001762 Ps001809
14.8 Ps001626
15.9 TI001I09
17.6 eSSR344-323
17.9 Ps001877
19.3 Ps001878 Ps001617
20.5 Ps000074
21.0 eSSR383-475
21.5 Ps900040 Ps900018 Ps900045
21.9 AC39-245
22.6 eSSR87-258
23.4 AA219-431
23.5 Sucsyn
28.4 AA174-442
29.2 Ps900010 TNI1F12
33.2 AA386-224
34.5 N13_600
37.2 Ps001554
37.4 AD186.2
38.3 Ps001550
39.2 Ps001795 Ps001761
39.8 Ps001907
41.0 Ps001680 Ps001904
41.5 Ps001895 Ps001632 Ps001777
42.2 Ps001636 Ps001567 Ps001828
44.7 Ps001705
45.3 eSSR470-301 Ps001725 Ps001899
45.7 Ps001757
46.2 eSSR48-497
46.8 Ps001127 Ps000906
47.1 eSSR2-312
48.0 Ps001655
48.1 O09_1900
48.6 eSSR73-418
48.9 Ps001596 Ps001553
49.1 Ps001889
49.2 Ps001695
49.3 eSSR425-523 Ps001743
50.8 Ps001317 Ps000304 Ps001854 eSSR446-504 Ps001718
51.5 Ps001919 Ps000789
51.7 Ps001748
53.9 Ps000559 Ps001871 Ps000731
54.6 Ps000215 Ps000481 Ps001540
55.3 Ps001911 Ps001548 Ps001686
56.0 TE002G15
56.9 Ps001372 Ps001818 Ps001896
57.5 Ps001799
57.8 TNE03L22
58.1 Ps001565 Ps001892 Ps001628
58.5 Ps001873
59.2 AB36-354
59.8 Ps001810 Ps001667 Ps001759 Ps001858 Ps001827
Ps001589
60.4 Ps001822
60.8 CE006N21
61.1 N14_1050
61.5 Ps001770
62.7 Ps001746
62.8 Ps001461
62.9 Ps001581 Ps001646 Ps001570 Ps001819
63.0 Ps001764 Ps001860 Ps001750 Ps001648
64.1 Ps001265 PA9-377 Ps001760 Ps001578 Ps001916
65.9 Ps001676 Ps001689
66.3 Ps001630
68.7 TE002H19
69.2 Ps001914 Ps001011
71.7 Ps000900 Ps900060 Ps900005
72.5 TI4A24 Ps900280 Ps900164
75.4 Ps001658
77.1 AA61-317
79.7 PSI1C24 eSSR281-386 eSSR57-334
80.5 AB128-245
81.3 Ps900217 AA92-386 U08_650 Ps900305 Ps900132
Ps900082 Ps900155
82.5 C07_700
87.9 Ps001881
89.4 Ps001606
89.9 Ps000512 Ps000816
90.4 Ps001742
91.8 Ps001814 Ps001876 TNE3A01a
93.1 AD186.b
93.7 PB16-436
95.8 F08_200
96.8 Ps001905 Ps001849
97.4 Ps001763
98.3 Ps001663 Ps000496
98.9 AA122-212
99.2 Ps001685
101.1 Y13.1950
101.2 Ps001723
101.6 Ps001744
103.3 Ps001464 CE007P08 Ps001417 Ps001069
103.8 Ps000975
104.7 CE006O20
105.2 Ps001724
105.4 Ps001605 Ps001622
106.6 Ps000086
107.4 Ps001857 Ps000111
107.8 Ps001805 Ps001696
108.0 Ps900097 Ps001621 Ps900174
108.8 TE002I24 AD53 S01.1800
110.0 Ps001714 TNE3A01c Ps001025 Ps000315
110.2 Ps001734 Ps001800 Ps001547 Ps001543 Ps001859
Ps001671
114.0 AB45
116.7 Ps001566 Ps001579
117.1 Ps001736 Ps001541 Ps001710
117.9 Ps001887
120.2 Ps000051
121.2 AD171-139
121.6 AC22-202
122.0 Ps000254
122.5 Ps001300
123.0 Ps000452
123.3 Ps001379
124.6 Ps001281
125.5 Ps001903
126.4 Ps000317
126.5 Ps900358 Ps900148 Ps900262
127.2 Ps001564
129.3 Ps000177
129.9 Ps000141
130.1 Ps000067
133.8 Ps001674
135.3 eSSR22-357
136.2 S04_750
139.3 AA349-158
141.6 Ps001591 eSSR389-405 Ps001679
143.0 TI001I06 BI2M15 Ps001749 Ps001839 Ps001866
Ps001708
143.9 Ps900301
145.2 Ps900024 Ps900265 Ps900188 eSSR459-300 Ps900081
Ps900271 Ps001619 Ps900292 Ps900027
146.1 Ps001574
147.3 Ps000700
148.3 Ps000631 Ps000556
149.5 AD61-134
152.9 AA315-359
155.7 Ps001613 Ps001870
156.5 Ps001664
157.1 Ps001898
158.3 Ps001555
159.2 Ps001780 eSSR478-188
160.0 eSSR477-307 Ps001491
160.4 Ps001610 Ps001657
161.8 Ps000842
162.3 Ps000216 Ps000203
162.6 Ps001057
162.7 Ps001635 Ps001592 Ps001584
163.4 Ps001868
164.5 Ps001758 Ps001456
165.9 Ps000815
168.2 Ps000928 Ps000935 Ps001043
168.8 PSVR
168.9 Ps000205
LGIV_BxPI_Duarte-derived
0.0 eSSR420-480
1.5 E12_1100
2.1 E12_900
10.3 Ps001690
16.3 Ps001878 Ps001617
17.9 Ps900045 Ps900018 Ps000074
18.5 AA219-431
19.5 Ps900040
20.2 AC39-245
23.3 AA174-442
26.1 AA386-224
27.5 AD186-314
30.0 Ps900248 Ps900083 Ps900035
32.5 Ps001907
33.8 Ps001904
36.4 N14_1900
36.5 Ps001828
40.8 Ps001725 Ps001899
44.2 Ps000906
45.0 O09_1900 eSSR73-418
45.6 Ps001596
48.3 Ps001718 Ps001919 Ps000789 Ps001317 eSSR446-504
Ps001748
49.1 AA285-365
51.6 Ps000731 Ps001871
52.0 Ps001911
52.4 Ps001818
53.5 Ps001799
54.0 Ps001892 Ps001810
54.1 Ps001628
54.2 Ps001873 Ps001827
54.3 Ps001589 Ps001759
54.4 AB36-354
55.6 Ps001770
56.8 Ps001822
58.1 Ps001746 Ps001860
58.7 Ps001265 Ps001916 PA9-377 Ps001760
59.9 Ps001676
66.2 Ps000900 Ps900005
68.5 Ps001658
70.7 AA61
71.9 AD249-341
73.1 eSSR281-386
73.4 AB128-245
74.8 AA92-386
75.4 Ps900305 Ps900132 Ps900217 Ps900082
80.1 Ps001881
81.7 Ps000512
82.3 Ps001742
83.3 PB16-436
84.7 Ps001849 Ps001905
86.0 Ps001663 AA122-212
87.3 Ps001744 Ps001723 Ps001417
87.9 Ps000975
89.5 Ps001605 Ps001724
95.6 AB45
98.7 AD53
100.2 AA378-415 Ps001857 Ps001800 Ps001805 Ps000315
Ps001714
100.8 Ps001579 Ps001566 Ps001543
101.4 Ps001541 Ps001710
103.9 AD171-139 Ps000254
109.5 Ps001281
110.0 Ps001903
110.6 Ps000317
113.3 Ps000141 Ps000067
119.7 eSSR22-357
123.9 AA349-158
126.2 Ps001591 Ps001679 eSSR389-405
127.9 Ps900301
128.9 Ps900024 Ps900265 Ps900081 Ps001619 Ps900271
Ps900292 Ps900027 Ps900188
129.9 Ps001574
130.4 Ps000700
131.6 AD61-134
133.4 AA315-359
134.9 Ps001613
135.4 Ps001664
136.4 Ps001898
138.4 Ps001491
138.9 Ps000842
139.4 Ps000216 Ps001584 Ps001635
141.0 Ps001868
141.9 eSSR419-195
145.1 Ps000205
145.7 Ps000935

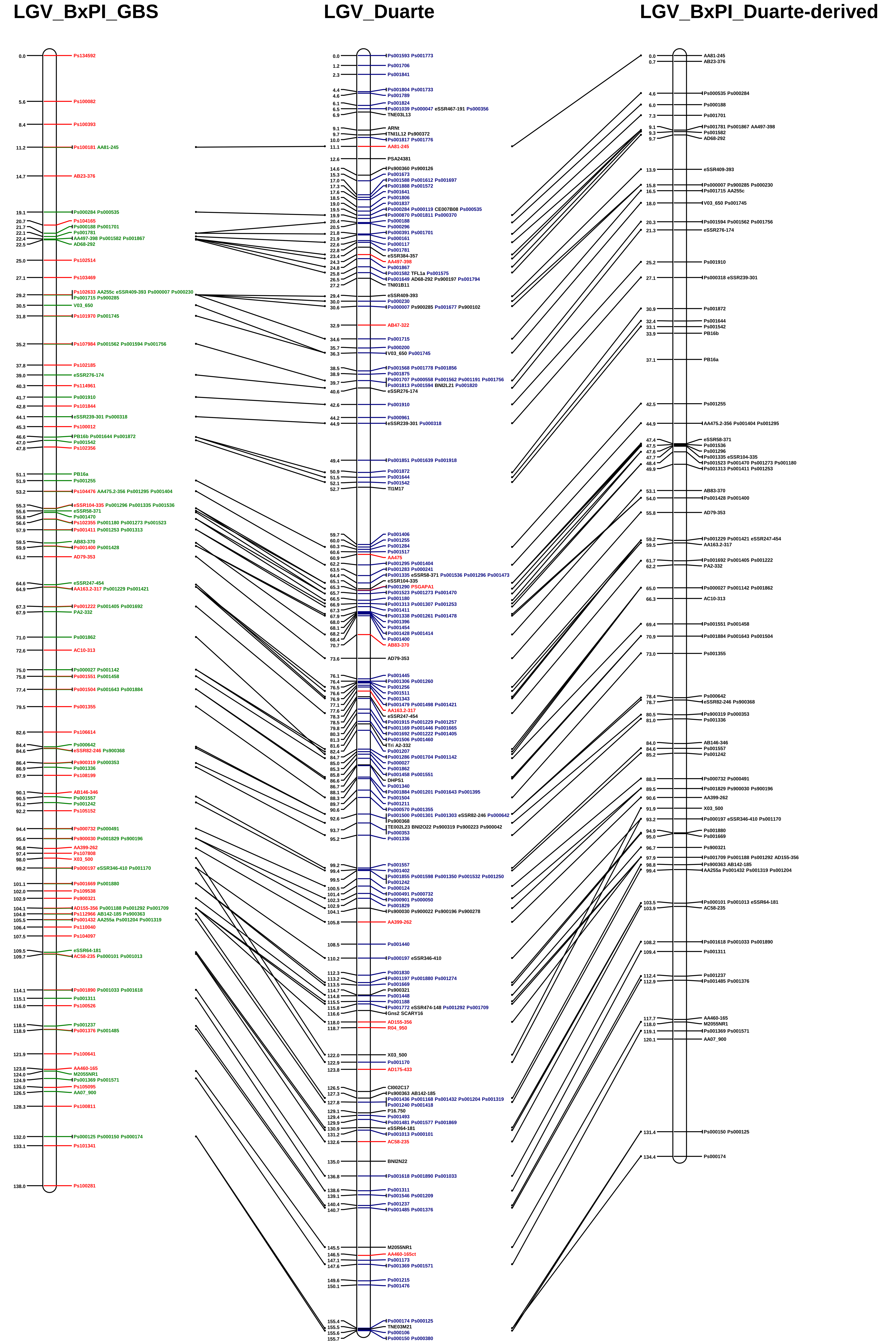
LGV_BxPI_GBS
LGV_Duarte
LGV_BxPI_Duarte-derived

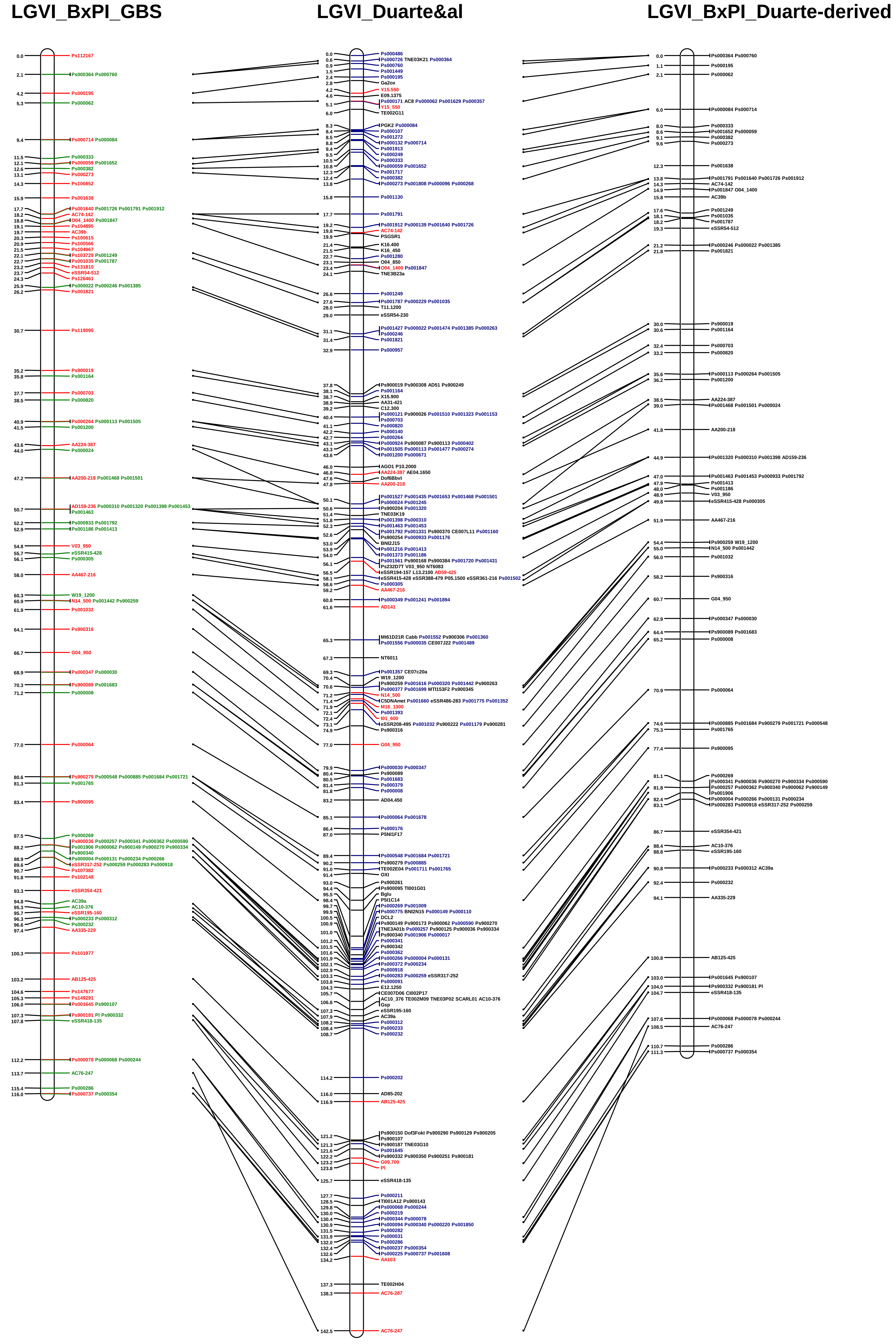
LGVI_BxPI_GBS
LGVI_Duarte&al
LGVI_BxPI_Duarte-derived

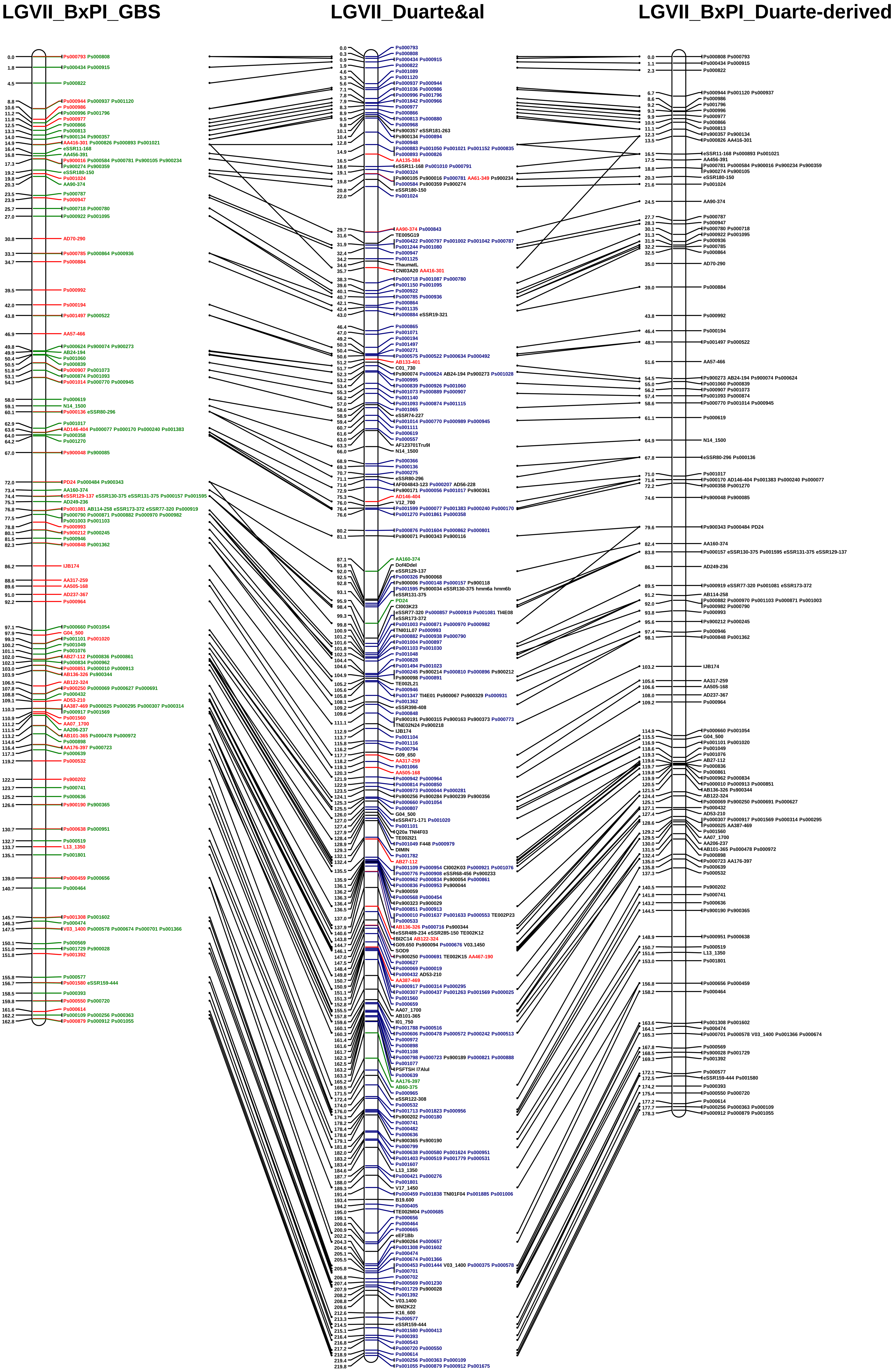
LGVII_BxPI_GBS
LGVII_Duarte&al
LGVII_BxPI_Duarte-derived
