# Supplementary material for: SNP discovery and genetic mapping using genotyping by sequencing of whole genome genomic DNA from a pea RIL population
Source: BMC Genomics. 2016 Feb 18;17:121. doi: 10.1186/s12864-016-2447-2 (PMC4758021; doi:10.1186/s12864-016-2447-2)
Supplement: Additional file 10: Table S7. — Parental accessions with partial resistance (PR), tolerance (TOL) or susceptibility (S) to stresses, used for sequencing and/or genotyping. (PDF 347 kb) [file 12864_2016_2447_MOESM10_ESM.pdf]

	Cultivated type	Sowing date	Aphanomyces	Ascochyta blight	Frost	Population	ref.
**'Baccara'**	Field	spring	S	S	S	Baccara'x'PI180693	Hamon *et al.* (2013)
**'PI180693'**	Ecotype		PR	unknown	unknown	Baccara'x'PI180693	Hamon *et al.* (2013)
**'552'**	Garden	spring	PR	S	S	Baccara'x'552'	Hamon *et al.* (2013)
**'JI296'**	Garden	spring	S	S	S	JI296'x'FP' ; 'JI296'x'DP'	Prioul *et al.* (2004)
**'FP'**	Field	winter	unknown	PR	TOL	JI296'x'FP'	Unpublished
**'DP'**	Fodder	winter	unknown	PR	TOL	'JI296'x'DP'	Prioul *et al.* (2004)
**'Terese'**	Field	spring	S	S	S	Champagne'x'Terese'	Lejeune-Hénaut *et al.* (2008)
**'Champagne'**	Fodder	winter	S	PR	TOL	Champagne'x'Terese'	Lejeune-Hénaut *et al.* (2008)

Hamon C, Coyne C, McGee R, Lesne A, Esnault R, Mangin P, Herve M, Le Goff I, Deniot G, Roux-Duparque M *et al*: **QTL meta-analysis provides a comprehensive view of loci controlling partial resistance to *Aphanomyces euteiches* in four sources of resistance in pea**. *BMC Plant Biology* 2013, **13**(1):45.

Lejeune-Hénaut I, Hanocq E, Béthencourt L, Fontaine V, Delbreil B, Morin J, Petit A, Devaux R, Boilleau M, Stempniak JJ, Thomas M, Lainé AL, Foucher F, Baranger A, Burstin J, Rameau C, Giauffret C : **The flowering locus *Hr* colocalizes with a major QTL affecting winter frost tolerance in *Pisum sativum* L.** *Theoretical and Applied Genet* 2008, **116** :1105-1116.

Prioul S, Frankewitz A, Deniot G, Morin G, Baranger A : **Mapping of Quantitative Trait Loci for partial resistance to *Mycosphaerella pinodes* in pea (*Pisum sativum* L.) at the seedling and adult plant stages**. *Theoretical and Applied Genet* 2004, **108** : 1322-1334.
